# Mediterranean diet, metabolic signature, genetic predisposition, and risk of rheumatoid arthritis: a large-scale population-based prospective cohort study

**DOI:** 10.1016/j.ajcnut.2025.09.051

**Published:** 2025-10-07

**Authors:** Xin Song, Xiaofeng Ma, Bin Yang, Di Zhang, Yanqiu Zou, Bowen Lei, Rong Xiang, Xunying Zhao, Yang Qu, Sirui Zheng, Ting Yu, Jinyu Zhou, Tao Han, Yangdan Zhong, Maoyao Xia, Lars Alfredsson, Karin Leander, Mengyu Fan, Xia Jiang

**Affiliations:** 1 Department of Epidemiology and Biostatistics, West China School of Public Health and West China Fourth Hospital, Sichuan University, Chengdu, Sichuan, China; 2 Department of Nutrition and Food Hygiene, West China School of Public Health and West China Fourth Hospital, Sichuan University, Chengdu, China; 3 Department of Clinical Neuroscience, Karolinska Institute, Stockholm, Sweden; 4 Institute of Environmental Medicine, Karolinska Institute, Stockholm, Sweden

**Keywords:** rheumatoid arthritis, Mediterranean diet, metabolic signature, genetic susceptibility, cohort study

## Abstract

**Background:**

Although the Mediterranean (MED) diet has been associated with reduced rheumatoid arthritis (RA) risk, the underlying metabolic mechanisms and the role of genetic susceptibility in this relationship remain unknown.

**Objectives:**

This study aims to identify a metabolic signature linked to the MED diet and examine its association with the risk of RA, while accounting for genetic predispositions.

**Methods:**

We analyzed data from 109,565 participants in the UK Biobank. Elastic net regression was applied to generate a MED-related metabolic signature. Cox proportional hazards models were used to assess the associations among MED diet score, its derived metabolic signature, and incident RA. A polygenic risk score for RA was incorporated to evaluate joint associations and potential interactions between genetic susceptibility and MED diet score or its metabolic signature in relation to RA risk. Mediation analysis was conducted to estimate the extent to which metabolic signature mediates the MED diet–RA association.

**Results:**

Over a median follow-up of 11.6 y, 1123 participants developed RA. We identified a MED diet-related metabolic signature comprising 66 metabolites. Both MED diet score and metabolic signature were inversely associated with RA risk—comparing the 90th to the 10th percentiles, hazard ratios for RA were 0.73 [95% confidence interval (CI): 0.63, 0.84] for MED diet score and 0.60 (95% CI: 0.50, 0.70) for metabolic signature. These associations remained consistent across all strata of genetic risk. Joint analyses indicated that favorable metabolic profiles may attenuate genetic predisposition to RA. Mediation analysis showed that the metabolic signature explained 22.4% (95% CI: 11.8%, 44.8%) of the MED diet–RA association.

**Conclusions:**

We identified a robust metabolic signature reflecting the metabolic response to the MED diet. This signature was inversely associated with RA risk and partially mitigated the genetic susceptibility to RA. These findings highlight the potential of metabolic signatures for enhancing dietary assessment and guiding personalized nutritional intervention.

## Introduction

Rheumatoid arthritis (RA) is a chronic inflammatory autoimmune disease affecting ∼0.5% of the global population [1]. The disease is characterized by joint pain, stiffness, swelling, fatigue, and progressive disability, frequently leading to reduced quality of life and increased risk of mortality [2]. Its etiology is believed to reflect a complex interplay between genetic susceptibility and environmental risk factors [3]. Among these, diet is considered a promising and modifiable target for prevention.

The 2022 American College of Rheumatology guideline recommends the Mediterranean (MED) diet as a cost-effective strategy for RA prevention [4]. This dietary pattern emphasizes high intake of fruits, vegetables, seafood, nuts, legumes, whole grains, and olive oil, moderate consumption of wine, and limited intake of red/processed meats, saturated fat, and sugary desserts or beverages [5]. A recent meta-analysis of 6 studies found that high adherence to the MED diet was associated with a 16% (7%, 24%) reduction in RA risk, potentially due to its anti-inflammatory and weight-reducing properties [6].

Although diet modulates disease risk through metabolic regulation [7], individual metabolic responses to identical diets exhibit substantial heterogeneity, of which the underlying mechanisms remain poorly understood. Traditional dietary assessment methods, such as food frequency questionnaires and dietary recalls, are limited by measurement errors and recall bias [8]. Metabolomics provides a powerful alternative, enabling objective and comprehensive quantification of both dietary intake and individualized metabolic responses [9]. The derived metabolic profiles reflect not only dietary intake but also interindividual variability influenced by genetic predisposition [10] and gut microbial activity [7]. Previous studies have identified an array of metabolites that are linked to specific foods, nutrients [[11], [12], [13]], and dietary patterns [[14], [15], [16]]. However, only 1 study to date has integrated questionnaire and metabolomic data to develop a lifestyle-related metabolic signature and evaluate its impact on RA risk. Although demonstrating partial mediation of lifestyle-related metabolic pathways, this study failed to assess specific dietary patterns or disentangle dietary effects from other lifestyle factors [17]. Therefore, 2 critical questions remain unresolved: *1*) whether the MED diet generates a distinctive metabolic signature, and *2*) whether such a signature predicts RA risk.

Genetic predisposition plays a major role in the onset of RA, accounting for ∼50% of disease susceptibility [18]. Emerging evidence suggests that lifestyle factors such as physical activity (PA), sleep patterns, and smoking can significantly attenuate this genetic predisposition [[19], [20], [21]]. However, it is unclear whether the MED diet similarly modifies genetic risk for RA. Moreover, the potential of integrating genetic and metabolomic data to enhance RA risk stratification and prediction accuracy remains unexplored.

Therefore, this study aims to identify a metabolic signature associated with the MED diet and evaluate its role in incident RA risk. Specifically, we first derived a metabolic signature using a machine learning approach based on 251 metabolomic biomarkers and a MED diet score derived from questionnaires. We then assessed the associations of this MED diet-related metabolic signature with RA risk, its interactions with genetic susceptibility, and its potential mediating role underlying the association between questionnaire-based MED diet score and RA.

## Patients and Methods

### Study population

We used data from the UK Biobank, an ongoing prospective cohort study, comprising over 500,000 participants aged 37–73 y at baseline, recruited between 2006 and 2010. Participants provided biological samples, completed touchscreen questionnaires, and underwent physical examinations. Details of the UK Biobank have been described previously [22]. All participants provided written informed consent, approved by the North West Multicenter Research Ethics Committee. Because the data were deidentified, no additional ethical approval was required for this analysis.

In this study, we included 210,944 participants who had completed ≥1 24-h online dietary questionnaire. We excluded individuals with extreme BMI (in kg/m^2^; < 15 or > 50, *n* = 843), implausible energy intake (< 500 or > 3500 kcal/d for females; < 800 or > 4200 kcal/d for males, *n* = 2594), RA diagnosis at baseline (*n* = 2386), missing metabolomic data (*n* = 94,462) or genetic data (*n* = 1094). Consequently, our final dataset comprised 109,565 participants (Supplemental Figure 1).

### Dietary assessment and MED dietary pattern

Participants who provided e-mail addresses were invited to complete the Oxford WebQ, a web-based 24-h dietary recall questionnaire covering 206 foods and 32 beverages, ≤5 occasions between April 2009 and June 2012 [23]. In this study, 39.4%, 22.9%, 20.4%, 14.6%, and 2.7% of participants completed 1, 2, 3, 4, and 5 questionnaires, respectively. A traditional MED diet was assessed using a validated MED diet score [5,24]. This score includes 13 dichotomous components: 9 recommended foods (vegetables, fruits, olive oil, white meat, wine, legumes, nuts, seafood, and Sofrito) and 4 limited foods (red and processed meat, butter and margarine, sugar-sweetened beverages, and sweets and desserts). Each component was assigned 1 point if intake met predefined cutoffs based on UK dietary recommendations and 0 otherwise. The total score was calculated as the sum of all components (range: 0–13), with higher values indicating better alignment with recommended dietary patterns (Supplemental Table 1). For each participant, dietary intake from available 24-h recalls (if multiple) was averaged to calculate an overall MED diet score.

### Metabolomics measurement

Metabolic profiling was conducted using nuclear magnetic resonance (NMR) spectroscopy by Nightingale Health on EDTA-treated plasma samples from ∼280,000 UK Biobank participants at baseline, with repeated metabolomic assessments performed in ∼16,000 individuals (referred to as the “first repeated assessment dataset”). Samples were analyzed in 2 phases: phase 1 (June 2019–April 2020) and phase 2 (April 2020–June 2022), using advanced NMR platforms at Nightingale Health’s facilities in Finland. All analyses followed standardized protocols and rigorous quality control, as previously described [14]. A total of 170 biomarkers were directly quantified, including lipids, fatty acids, and 81 derived metabolite ratios, covering a broad range of metabolic pathways, such as detailed lipoprotein subclass-specific lipid profiling. In total, 251 metabolic biomarkers were included in the analysis (Supplemental methods and Supplemental Table 2).

### Ascertainment of outcome

Incident RA cases (International Classification of Diseases, 10th Revision (ICD-10) codes: M05, M06) were identified using UK Biobank electronic health records (Field IDs: 131848-131851), including hospital admissions, primary care, death registers, and self-reported conditions [25]. The availability of electronic health records varied by region, with data accessible up to 31 October, 2022, for England, 31 August, 2022, for Scotland, and 31 May, 2022, for Wales. Person-years were calculated from the date of the participant’s first completed 24-h online dietary questionnaire to the earliest of the following events: incident RA diagnosis, death, loss to follow-up, or end of follow-up.

### Polygenic risk score for RA

The polygenic risk score (PRS) for RA was derived from validated “Standard PRS (Category 301)” provided in the UK Biobank Release [26,27]. The PRS was calculated by summing effect sizes of genetic variants multiplied by their allele dosages, using a Bayesian methodology applied to meta-analyzed genome-wide association study (GWAS) summary statistics. Higher scores indicated a higher genetic predisposition to RA. In this study, participants were categorized into low (quintile 1), intermediate (quintiles 2–4), and high (quintile 5) genetic risk groups based on their position in the PRS distribution.

### Ascertainment of covariates

Potential covariates were selected at baseline based on previous literature and expert knowledge [6,17]. Covariates included age (continuous, years), sex (male or female), ethnicity (White or non-White), assessment center (22 centers), Townsend deprivation index (TDI, continuous), educational attainment (university degree or other), PA (continuous, metabolic equivalent hours/week), smoking status (never, ever, or current), sleep duration (continuous, hours/day), energy intake (continuous, kcal/day), multivitamin use (yes or no), fasting duration (continuous, hours/day), BMI (continuous), prevalent hypertension, hyperlipidemia, diabetes, cardiovascular disease, and cancer at baseline, the top 10 genetic principal components (PCs), and genotype batch. In sensitivity analyses, we additionally adjusted for use of nonsteroidal anti-inflammatory drugs (NSAIDs), presence of other autoimmune diseases, and waist circumference.

### Statistical analysis

Missing data were handled through mean imputation for continuous variables and creation of separate indicator categories for categorical variables. Baseline characteristics of participants across quintiles of the MED diet score were described as means ± SD for continuous variables and percentages for categorical variables.

All 251 metabolite concentrations were log-transformed to normalize distributions and subsequently standardized as *z*-scores. A 2-stage analytical approach was applied to derive a metabolic signature linked to the MED diet. Initially, a multivariable-adjusted linear regression model was used to assess the association between each metabolite and the MED diet score, applying a Bonferroni correction for multiple testing (*P* < 0.05/251). Subsequently, metabolites showing significant associations were entered into an elastic net regression using the *R* package “glmnet” for biomarker selection [28]. Participants were randomly divided into training (70%) and testing (30%) datasets. For each mixing parameter *α* (ranging from 0 to 1 in increments of 0.05), the regularization parameter *λ* was optimized in the training dataset via 10-fold cross-validation using 1 one SE rule (lambda.1se), and predictive performance was subsequently evaluated in the testing dataset. The optimal value (*α* = 0.95) was selected based on the lowest mean squared error, and metabolic signature was calculated as a weighted sum of selected metabolites, with weights derived from elastic net regression coefficients. To validate the performance of the established metabolic signature, parameters from the trained model were applied to the testing dataset as well as to the first repeated assessment dataset. Leave-one-out cross-validation was further applied in the training dataset to evaluate model robustness.

Cox proportional hazards models were used to estimate the hazard ratios (HRs) with 95% confidence intervals (CIs) for the associations of MED diet score, metabolic signature, and individual metabolites with the risk of RA. The MED diet score and metabolic signature were analyzed as quintile-based categorical variables and as standardized continuous variables (per increment from the 10th to the 90th percentile). The proportional hazards assumption was tested using Schoenfeld residuals with no violation detected. To address potential confounding, 3 models were established where model 1 was adjusted only for age and sex; model 2 was additionally adjusted for assessment center, TDI, educational attainment, PA, smoking status, sleep duration, energy intake, multivitamin use, fasting duration, BMI, hypertension, hyperlipidemia, diabetes, cardiovascular disease, cancer, PRS for RA, the first 10 genetic PCs and genotype batch; model 3 was mutually adjusted for both the MED diet score and the metabolic signature based on model 2 to evaluate their independent associations with RA risk. Dose–response associations were assessed using restricted cubic splines with knots at the 10th, 50th, and 90th percentiles. Stratified analyses were performed by age, sex, TDI, educational attainment, smoking status, PA, sleep duration, and BMI. Interactions of the MED diet score or metabolic signature with these stratification variables were tested using likelihood ratio tests.

We further examined the joint association of the MED diet score, metabolic signature, and PRS with the risk of RA, with the reference group being the highest-risk participants, that is, those with the lowest MED diet score or metabolic signature and the highest PRS. Multiplicative interactions were assessed by comparing models with and without an interaction term. In addition, we evaluated additive interactions to further explore the potential joint effects of exposures. Measures of additive interaction were estimated using relative excess risk due to interaction, attributable proportion due to interaction, and synergy index, with corresponding 95% CIs derived using the delta method. Additional stratified analyses were conducted across PRS strata to assess the associations of the MED diet score or metabolic signature with RA risk.

To investigate associations potentially mediated through the metabolic signature or individual metabolites, mediation analyses were conducted using the R package “CMAverse” [29]. Linear regression was used for the exposure–mediator associations, whereas Cox proportional hazards models were applied for the mediator–outcome associations. Indirect, direct, and total effects for each mediator were estimated by integrating the exposure–mediator and mediator–outcome models. Nonparametric bootstrap resampling was used to calculate 95% CIs for the proportion mediated. Candidate mediators are ideally selected based on their significant associations with both the exposure and the outcome, after the temporal sequence in which exposure precedes the mediator and the mediator precedes the outcome [30]. However, given the cross-sectional nature of our dietary and metabolomic measurements, the temporal assumption cannot be established. Therefore, candidate mediators were selected solely based on their significant associations with both the exposure and the outcome.

To assess the robustness of results, multiple sensitivity analyses were conducted. First, additional adjustments were made for NSAIDs use and baseline autoimmune comorbidities. Second, waist circumference was included to account for potential confounding by central adiposity. Third, analyses were restricted to participants with complete data. Fourth, individuals with fewer than 2 dietary assessments were excluded to improve the reliability of exposure measurement. Fifth, cases occurring within the initial 2 y of follow-up were excluded to mitigate reverse causation. Sixth, individuals who reported a “nontypical diet yesterday” were excluded. Seventh, the competing risk of death was addressed using Fine and Gray’s subdistribution hazard models. Finally, each component of the MED diet score was sequentially removed to reassess its contribution to RA risk.

Analyses were performed using R software (version 4.4.0). Statistical significance was defined as a 2-tailed *P* value < 0.05.

## Results

### Characteristics of the study participants

A total of 109,565 participants were included in the analysis, with a median follow-up of 11.6 y and contributing 1,255,665 person-years. During the follow-up, 1123 incident cases of RA were identified. Table 1 summarizes baseline characteristics across quintiles of the MED diet score. Among all participants, the mean age was 58.4 y (SD = 8.0), the mean MED diet score was 4.8 (SD = 1.7), and 49,972 (45.6%) were males. Compared with those in the lowest quintile of the MED diet score, participants in the highest quintile were more likely to be older, females, and have higher educational attainment and socioeconomic status. They also tended to engage more in PA, be less likely to be current smokers, have healthier BMI, report greater multivitamin use, and have lower prevalence of baseline comorbidities.

**TABLE 1:** Baseline characteristics of the study participants according to quintiles of the MED diet score^1^.

Characteristics	All	Quintile 1	Quintile 2	Quintile 3	Quintile 4	Quintile 5
No. of participants	109,565	26,387	22,996	24,288	18,689	17,205
Dietary score	4.75 (1.74)	2.50 (0.70)	4.00 (0.00)	5.00 (0.00)	6.00 (0.00)	7.51 (0.77)
Age (y)	58.36 (7.99)	57.88 (8.16)	58.23 (8.10)	58.41 (7.97)	58.70 (7.85)	58.86 (7.72)
Male	49,972 (45.61)	14,425 (54.67)	10,850 (47.18)	10,622 (43.73)	7569 (40.50)	6506 (37.81)
White	105,219 (96.03)	25,539 (96.79)	22,161 (96.37)	23,272 (95.82)	17,857 (95.55)	16,390 (95.26)
TDI	−1.67 (2.84)	−1.55 (2.90)	−1.70 (2.83)	−1.74 (2.81)	−1.78 (2.77)	−1.61 (2.88)
College degree	46,217 (42.18)	8531 (32.33)	8923 (38.80)	10,689 (44.01)	9088 (48.63)	8986 (52.23)
Physical activity METs (h/wk)	41.73 (36.91)	40.73 (38.11)	40.99 (36.89)	40.88 (35.95)	42.37 (35.64)	44.74 (37.61)
Current smoker	8356 (7.63)	2653 (10.05)	1808 (7.86)	1707 (7.03)	1174 (6.28)	1014 (5.89)
Sleep (h/d)	7.17 (1.00)	7.16 (1.05)	7.17 (1.02)	7.17 (0.99)	7.18 (0.98)	7.17 (0.96)
Energy (kcal/d)	2048.11 (551.28)	2107.75 (568.06)	2029.96 (552.28)	2013.92 (542.28)	2017.11 (535.10)	2062.84 (545.81)
Multivitamin users	20,096 (18.34)	4102 (15.55)	4135 (17.98)	4530 (18.65)	3582 (19.17)	3747 (21.78)
Fasting duration (h)	3.65 (2.11)	3.70 (2.24)	3.66 (2.14)	3.63 (2.08)	3.64 (2.02)	3.61 (2.04)
BMI (kg/m^2^)	26.94 (4.54)	27.89 (4.84)	27.21 (4.59)	26.75 (4.38)	26.41 (4.32)	25.95 (4.12)
Individual history of disease						
Hypertension	9596 (8.76)	2744 (10.40)	2117 (9.21)	2021 (8.32)	1476 (7.90)	1238 (7.20)
Hyperlipidemia	3904 (3.56)	1068 (4.05)	904 (3.93)	851 (3.50)	590 (3.16)	491 (2.85)
Diabetes	1930 (1.76)	683 (2.59)	442 (1.92)	405 (1.67)	240 (1.28)	160 (0.93)
Cardiovascular disease	3154 (2.88)	885 (3.35)	688 (2.99)	693 (2.85)	500 (2.68)	388 (2.26)
Cancer	11,454 (10.45)	2729 (10.34)	2389 (10.39)	2476 (10.19)	1973 (10.56)	1887 (10.97)

*Abbreviations:* MED, Mediterranean; METs, metabolic equivalent for task score; TDI, Townsend deprivation index.

1 Values are means ± SD for continuous variables and *N* (%) for categorical variables.

### Identification of the MED diet-related metabolic signature

**FIGURE 1:**
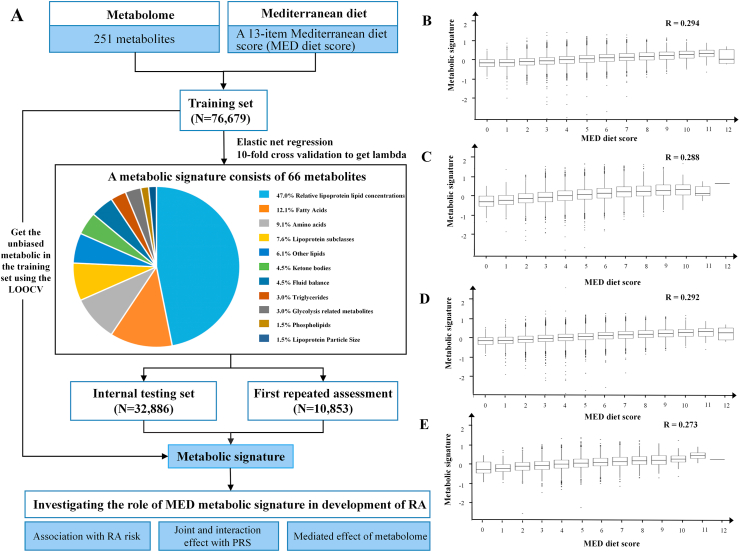
Flowchart of study design and associations between the metabolic signature and MED diet score. (A) The training and testing procedures of a metabolic signature for the MED diet score. Correlation between the MED diet score and (B) the corresponding metabolite signature score in the training set (C), the corresponding metabolite signature score in the testing set (D), the corresponding metabolite signature score in the combined data (E), and the corresponding metabolite signature score in the first repeat assessment data. Pearson correlation is employed in correlation analysis. A 2-sided *P* < 0.05 was considered statistically significant. MED, Mediterranean; LOOCV, leave-one-out cross-validation; PRS, polygenic risk score; RA, rheumatoid arthritis.

High pairwise correlations were observed among the 251 metabolites (Supplemental Figure 2). To derive a metabolic signature linked to the MED diet score, a 2-step approach was implemented. In the initial multivariable linear analysis, 193 (76.9%) metabolites were significantly associated with the MED diet score at baseline (Supplemental Table 3), including lipoprotein subclasses (*n* = 72), relative lipoprotein lipid concentrations (*n* = 60), fatty acids (*n* = 14), and the others (*n* = 47). Subsequently, elastic net regression was conducted using these 193 metabolites to construct a metabolic signature and address multicollinearity. This procedure identified 66 metabolites most strongly associated with the MED diet score (Figure 1 and Supplemental Table 4), including relative lipoprotein lipid concentrations (*n* = 31), fatty acids (*n* = 8), amino acids (*n* = 6), lipoprotein subclasses (*n* = 5), and the others (*n* = 16). The 66-metabolite signature showed consistent significant correlations with MED diet scores across different datasets: the training (*r* = 0.294, *P* < 0.001), the testing (*r* = 0.288, *P* < 0.001), the combined (*r* = 0.292, *P* < 0.001), and the first repeated assessment (*r* = 0.273, *P* < 0.001) (Figure 1). Similar performance was observed in leave-one-out cross-validation within the training dataset (*r* = 0.288, *P* < 0.001).

**FIGURE 2:**
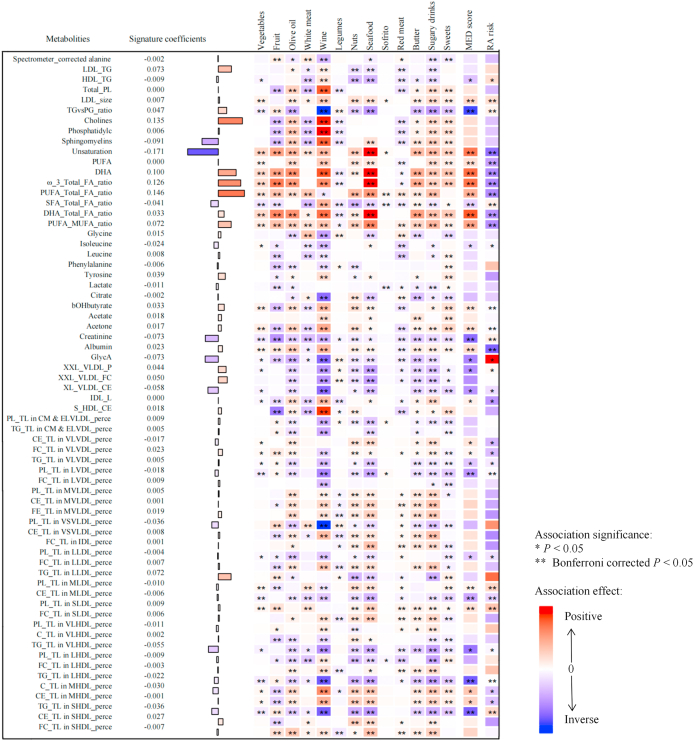
Associations of the 66 metabolites constituting the metabolic signature with MED components, total MED diet score, and subsequent RA risk. Presented from left to right are the metabolites’ coefficients (weights) in the signature, associations with each food component, total MED diet score, and subsequent RA risk. Coefficients for associations with food items and total MED diet score indicate the SD changes in metabolites per SD increment in dietary intake. Coefficients for RA risk indicate hazard ratio of RA disease risk per SD increment in metabolites. The weights in the signature were evaluated using elastic net regression. Linear regressions were used to analyze the connection among metabolites, food components, and the total MED diet score. Cox regressions were utilized to analyze the association between metabolites and the risk of RA. All models were adjusted similarly to model 2 in Table 2. Colors indicate the direction (red for positive and blue for inverse) and strength (darker color for stronger magnitude) of the associations; asterisks denote significance (∗ *P* < 0.05 and ∗∗ Bonferroni corrected *P* < 0.05; for associations with total MED diet score and RA risk, we Bonferroni corrected for 66 metabolites; for associations with each food item, we Bonferroni corrected for 66 metabolites × 13 food items). Abbreviations for metabolites are provided in Supplemental Table 2. MED, Mediterranean; RA, rheumatoid arthritis.

Notably, metabolites positively correlated with the MED diet score, such as ratio of DHA to total fatty acids, DHA, ratio of omega-3 (n–3) fatty acids to total fatty acids, and degree of unsaturation, were also positively associated with the intake of foods like olive oil, wine, and seafood. Conversely, metabolites negatively correlated with the MED diet score, such as ratio of triglycerides to phosphoglycerides, triglycerides to total lipids in small HDL percentage, triglycerides to total lipids in large HDL percentage, and creatinine were positively associated with the consumption of butter, sugary drinks, and sweets. Among the 66 metabolites, ratio of DHA to total fatty acids, DHA, ratio of n–3 fatty acids to total fatty acids, and degree of unsaturation emerged as the most significant biomarkers associated with the MED diet score (Figure 2).

### Associations of MED diet score, metabolic signature with the risk of RA

Participants in the highest quintile of the MED diet score (Q5) showed a 32% reduced risk of RA (HR: 0.68; 95% CI: 0.55, 0.84) compared with the lowest quintile (Q1) and in a dose–response manner (*P*_trend_ < 0.001). A similar yet more pronounced association was observed for the metabolic signature (Q5 compared with Q1: HR: 0.55; 95% CI: 0.44, 0.68). When mutually adjusted, both MED diet score (HR: 0.75; 95% CI: 0.60, 0.92) and metabolic signature (HR: 0.58; 95% CI: 0.47, 0.73) were independently associated with RA risk. Comparing the 90th to the 10th percentile, results remained consistent for MED diet score (HR: 0.73; 95% CI: 0.63, 0.84) and for metabolic signature (HR: 0.60; 95%CI: 0.50, 0.70) (Table 2). Restricted cubic spline analysis confirmed linear associations (*P*_nonlinearity_ = 0.445 for MED diet score; *P*_nonlinearity_ = 0.565 for metabolic signature) (Supplemental Figure 3). In the first repeated measurement dataset, the association between the metabolic signature and incident RA remained directionally consistent and of similar magnitude (HR: 0.63; 95% CI: 0.38, 1.02) (Supplemental Table 5).

**TABLE 2:** Association of the MED diet score and metabolic signature with RA risk^1^.

	Quintile 1	Quintile 2	Quintile 3	Quintile 4	Quintile 5	Comparing the 90th to 10th percentiles	*P* for trend^2^
MED diet score							
No. of RA cases	310	267	242	171	133	1123	
Person-years	300,648	263,189	279,056	214,787	197,985	1,255,665	
Incidence/100,000 PYs	103.11	101.45	86.72	79.61	67.18	89.43	
Model 1	1.00 (ref)	0.93 (0.79, 1.10)	0.78 (0.66, 0.92)	0.70 (0.58, 0.84)	0.58 (0.47, 0.71)	0.63 (0.55, 0.73)	<0.001
Model 2	1.00 (ref)	1.00 (0.84, 1.17)	0.86 (0.73, 1.02)	0.79 (0.66, 0.96)	0.68 (0.55, 0.84)	0.73 (0.63, 0.84)	<0.001
Model 2+mutual adjustment	1.00 (ref)	1.02 (0.87, 1.21)	0.90 (0.76, 1.07)	0.85 (0.70, 1.03)	0.75 (0.60, 0.92)	0.78 (0.68, 0.91)	0.002
Metabolic signature							
No. of RA cases	286	244	226	196	171	1123	
Person-years	248,546	250,835	251,864	252,104	252,315	1,255,665	
Incidence/100,000 PYs	115.07	97.28	89.73	77.75	67.77	89.43	
Model 1	1.00 (ref)	0.75 (0.63, 0.89)	0.62 (0.52, 0.75)	0.48 (0.4, 0.58)	0.40 (0.33, 0.48)	0.45 (0.39, 0.52)	<0.001
Model 2	1.00 (ref)	0.83 (0.70, 0.99)	0.76 (0.63, 0.92)	0.62 (0.51, 0.76)	0.55 (0.44, 0.68)	0.60 (0.50, 0.70)	<0.001
Model 2+mutual adjustment	1.00 (ref)	0.84 (0.71, 1.00)	0.78 (0.65, 0.94)	0.64 (0.52, 0.78)	0.58 (0.47, 0.73)	0.63 (0.53, 0.75)	<0.001

Model 1 was adjusted for age and sex.

Model 2 was additionally adjusted for assessment center, Townsend Deprivation Index, educational attainment, physical activity, smoking status, sleep duration, energy intake, multivitamin use, fasting duration, BMI, hypertension, hyperlipidemia, diabetes, cardiovascular disease, cancer, polygenic risk score for rheumatoid arthritis, the top 10 genetic principal components, and genotype batch.

Model 2 + mutual adjustment further included both the MED diet score and its metabolic signature to assess their independent associations.

Abbreviations*:* MED, Mediterranean; PYs, person-years; RA, rheumatoid arthritis.

1 Data are hazard ratios (95% confidence intervals).

2 *P* for trend was calculated across quintiles using multivariable Cox regression models. A 2-sided *P* < 0.05 was considered statistically significant.

### MED diet score, metabolic signature, genetic risk, and RA incidence

**FIGURE 3:**
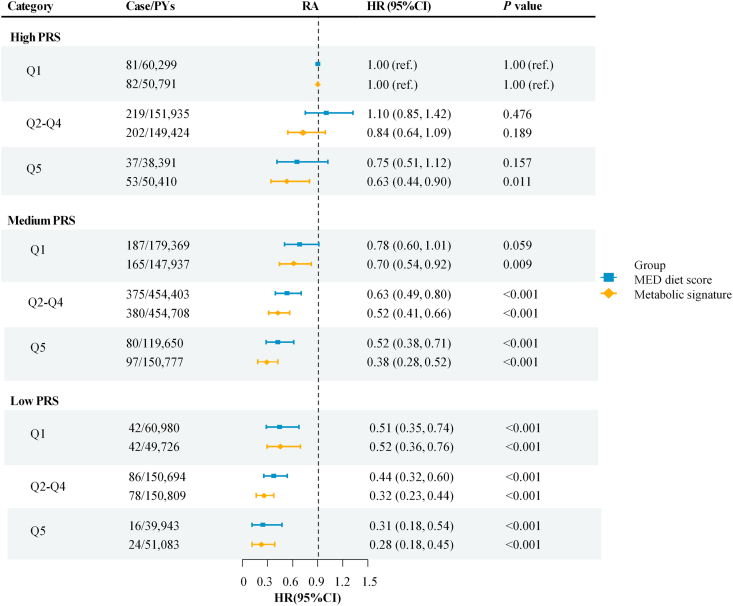
Joint association of the MED diet score, metabolic signature, and genetic susceptibility with risk of RA. Adjusted for age, sex, assessment center, Townsend Deprivation Index, educational attainment, physical activity, smoking status, sleep duration, energy intake, multivitamin use, fasting duration, BMI, hypertension, hyperlipidemia, diabetes, cardiovascular disease, cancer, the top 10 genetic principal components, and genotype batch. CI, confidence interval; HR, hazard ratio; MED, Mediterranean; PRS, polygenic risk score; PYs, person-years; RA, rheumatoid arthritis.

Participants with a high PRS showed a significantly increased risk of RA compared with those with a low PRS (HR: 2.33; 95% CI: 1.92, 2.84) (Supplemental Table 6). Joint analyses showed that individuals with low PRS and the highest quintile of the MED diet score had the lowest risk of RA (HR: 0.31; 95% CI: 0.18, 0.54) compared with the reference group (high PRS and the lowest quintile of the MED diet score). A similar but even stronger protective association was observed for metabolic signature (HR: 0.28; 95% CI: 0.18, 0.45) (Figure 3). Notably, participants with a high PRS but the highest quintile of the MED diet score (HR: 0.75; 95% CI: 0.51, 1.12) had a similar risk of RA as those with a medium PRS but the lowest quintile of the MED diet score (HR: 0.78; 95% CI: 0.60, 1.01). Similarly, participants with a high PRS but a favorable metabolic signature (HR: 0.63; 95% CI: 0.44, 0.90) had a comparable or even slightly lower RA risk than those with a medium PRS but an unfavorable metabolic signature (HR: 0.70; 95% CI: 0.54, 0.92).

Although both MED diet score and metabolic signature were inversely associated with RA risk across PRS strata, the associations were more pronounced for metabolic signature (Supplemental Tables 7 and 8). These findings suggest the metabolic signature captures individual dietary responses to a better extent than self-reported dietary patterns, and confers protection against RA even among genetically predisposed individuals. No significant multiplicative or additive interactions were observed between the MED diet score or metabolic signature and PRS (Supplemental Table 9).

### Mediation analysis

**FIGURE 4:**
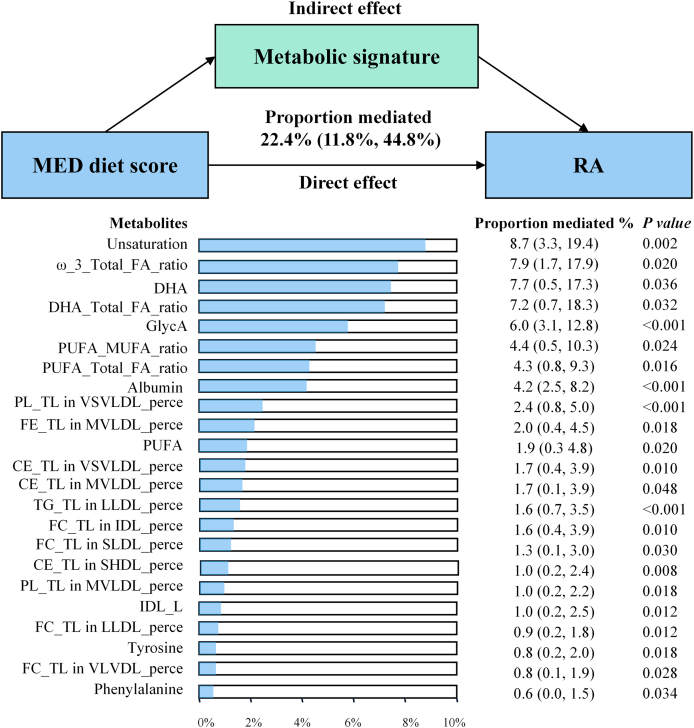
Association of the MED diet score with RA mediated by metabolic signature and metabolites. Adjusted for age, sex, assessment center, Townsend Deprivation Index, educational attainment, physical activity, smoking status, sleep duration, energy intake, multivitamin use, fasting duration, BMI, hypertension, hyperlipidemia, diabetes, cardiovascular disease, cancer, the top 10 genetic principal components, and genotype batch. Abbreviations for metabolites are provided in Supplemental Table 2. MED, Mediterranean; RA, rheumatoid arthritis.

Metabolic signature was estimated to mediate 22.4% (95% CI: 11.8%, 44.8%) of the MED diet score-RA association. Additionally, 22 individual metabolites were significant mediators, each explaining 0.6%–8.7% of the association. Notably, several lipid-related biomarkers including degree of unsaturation, ratio of omega-3 fatty acids to total fatty acids, DHA, and ratio of DHA to total fatty acids showed the highest proportions of mediation, ranging from 7.2% to 8.7% (Figure 4).

### Subgroup and sensitivity analysis

In subgroup analysis, the inverse associations of both the MED diet score and metabolic signature with RA risk were more pronounced among participants who engaged in moderate-to-vigorous PA, had healthy sleep durations (7–8 h/night), and had a BMI < 30 (Supplemental Figure 4). Multiple sensitivity analyses confirmed the robustness of results. Sequential removal of individual MED diet components minimally altered the overall diet–RA association (Supplemental Table 10). Additional adjustments for potential confounders, including NSAIDs use, other autoimmune diseases, and waist circumference, did not change the results. The associations remained consistent when analyses were restricted to participants with complete data, with multiple dietary assessments, when excluding cases occurring within the initial 2 y of follow-up, when excluding participants with atypical dietary reporting, and when employing competing risk models accounting for mortality (Supplemental Table 11).

## Discussion

Leveraging data from a large-scale population-based prospective cohort, we identified a metabolic signature associated with the MED diet. This metabolic signature demonstrated a stronger inverse association with RA risk than the MED diet score, even among genetically predisposed individuals. Moreover, this metabolic signature partially mediated the observed protective association between the MED diet score and RA risk.

Although previous studies have linked MED diet-related metabolic signatures to reduced risks of cardiovascular disease, diabetes, and frailty [[14], [15], [16]], their potential role in RA prevention remains unclear. The sole existing study linking metabolic profiles to RA considered overall lifestyle factors without distinguishing specific dietary contributions [17]. To address this gap, we derived a 66-metabolite signature reflecting MED diet and further evaluated its association with incident RA. This signature predominantly included lipid metabolism biomarkers, aligning with the MED diet’s emphasis on unsaturated fats [14]. Higher intake of key MED components—olive oil, seafood, and wine—was associated with elevated levels of beneficial lipid biomarkers, such as DHA and omega-3 PUFAs, with anti-inflammatory and antioxidant properties [[31], [32], [33]]. These lipids, especially DHA, may modulate immune responses in RA pathogenesis through several mechanisms, including suppressing proinflammatory eicosanoid synthesis, promoting specialized proresolving mediators, and inhibiting nuclear factor-κB (NF-κB) activation in immune cells [34]. Conversely, metabolites inversely associated with MED diet correlated with unhealthy food consumption [35,36]. Among these, glycoprotein acetyls (GlycA), a NMR marker of glycosylated acute-phase proteins and systemic inflammation, was noted. Elevated GlycA levels have been consistently associated with adverse lipid remodeling, reduced insulin sensitivity, and heightened risk of cardiometabolic and autoimmune diseases [37,38]. Our findings suggest that the MED diet may lower RA risk by beneficially modulating lipid metabolism, whereas poor dietary quality promotes proinflammatory metabolic profiles. Our results highlight metabolomic regulation as a key mechanism linking diet to autoimmune disease risk, supporting the potential of dietary interventions in RA prevention.

Growing evidence suggests the plasma metabolome serves as a comprehensive health indicator, integrating influences of diets, genetics, gut microbiota, and overall health conditions [7,10]. Our study showed that the MED-related metabolic signature effectively captures the systemic biological response to dietary patterns. By offering more objective measurements than dietary questionnaires, metabolic profiles complement conventional nutritional assessments and enable personalized interventions. The protective associations of MED diet and its metabolic signature with RA were stronger among participants with healthier lifestyles, suggesting optimal lifestyles may potentiate the metabolic benefits of diet through improved metabolic flexibility and reduced inflammation. Regular PA and adequate sleep duration, both established modifiers of inflammatory pathways and metabolic homeostasis, appear to amplify the favorable metabolic responses linked to the MED diet [19]. Conversely, the proinflammatory characteristics of obesity may counteract the beneficial metabolic responses associated with diet [39]. Collectively, these findings emphasize multimodal RA prevention strategies that combine high-quality diet patterns, regular PA, adequate sleep, and weight management to optimize metabolic health and reduce autoimmune disease risk.

Favorable metabolic profiles were consistently associated with decreased RA risk across all strata of genetic predisposition, including among those high-risk individuals with elevated PRS. This suggests that optimal metabolic health may partially offset genetic susceptibility to RA, although no significant interaction was observed between metabolic profiles and PRS. Future studies with larger number and diverse genetic backgrounds are needed to validate these observations and elucidate the interplay among dietary exposures, metabolic responses, and genetic factors in RA development.

This study has several important strengths. It leverages a large, well-characterized population cohort with extended follow-up, enabling robust assessment of incident RA cases. Comprehensive metabolic profiling provides objective insights into diet-related biological responses, and integration of genetic data enables evaluating the interplay among diet, metabolic signatures, and genetic susceptibility in RA risk. Nonetheless, several limitations should be acknowledged. First, some participants provided only 1 24-h dietary recall questionnaire, potentially limiting assessment of typical consumption patterns and introducing measurement imprecision. However, previous studies have reported moderate to substantial agreement between baseline and repeat assessments of 24-h dietary recall questionnaires [23]. Moreover, sensitivity analyses excluding participants with only a single recall yielded consistent results. Second, despite 251 metabolites being assessed, the metabolomics platform did not capture the complete spectrum of diet-related biomarkers. Third, the single-timepoint metabolomic assessment may not fully account for biological variability, though repeated assessments suggest reasonable temporal stability for most metabolites over 1–2 y. Fourth, the predominantly self-reported White composition (96.03%) of the UK Biobank may restrict the applicability of findings to ethnically diverse populations. Fifth, the observational design to some degree limits our ability to make causal inferences among MED diet, the associated metabolic profiles, and the risk of RA. Finally, because both dietary intake and metabolic profiling were assessed cross-sectionally, temporality cannot be firmly established. Therefore, the observed mediation role of the metabolic signature in the MED diet–RA association should be interpreted cautiously and may reflect shared biological pathways linking MED diet, metabolic changes, and RA risk, rather than a sequential causal pathway. Future longitudinal studies with repeated measurements are needed to clarify these temporal relationships.

In summary, our investigation identifies a distinctive metabolic signature associated with the MED diet that is inversely related to the risk of developing RA. The 66-metabolite profile not only serves as an objective biomarker of dietary pattern but also elucidates key biological mechanisms mediating the MED diet–RA relationship. By integrating information on both dietary compliance and individual metabolic response, this signature advances beyond traditional assessment methods, enabling improved risk stratification incorporating genetic and lifestyle factors, and targeted nutritional interventions tailored to metabolic profiles. The demonstrated stability of these metabolic associations across genetic risk strata further underscores their potential clinical utility in the general population.

## Author contributions

The authors’ responsibilities were as follows – XS, XM, MF, XJ: conceptualized the study and wrote the manuscript; XS, XM: performed the primary analyses, with assistance from BY, DZ, BL, YZ (statistical analysis), RX, XZ, YQ, SZ, TY, JZ, TH, YZ, MX (result interpretation); LA, KL, LK: provided critical suggestions for the manuscript writing; XJ, MF: supervised the study; and all authors: contributed to the interpretations of the findings, the critical revision of the manuscript, and read and approved the final manuscript.

## Data availability

The genetic and phenotypic UK Biobank data are available on application to the UK Biobank to any researcher worldwide (www.ukbiobank.ac.uk).

## Funding

This study was supported by the Science Fund for Creative Research Groups of Science and Technology Bureau of Sichuan Province (2024NSFTD0030), the Recruitment Program for Young Professionals of China, the Promotion Plan for Basic Medical Sciences and the Development Plan for Cutting-Edge Disciplines, Sichuan University, the Experimental Discipline Revitalization Plan of West China School of Public Health and West China Fourth Hospital (2023SY-03), and other Projects from West China School of Public Health and West China Fourth Hospital, Sichuan University. The sponsors of this study had no role in study design, data collection, analysis, interpretation, writing of the report, or the decision for submission.

## Conflict of interest

The authors report no conflicts of interest.
