# Supplementary material for: Mediterranean diet, metabolic signature, genetic predisposition, and risk of rheumatoid arthritis: a large-scale population-based prospective cohort study
Source: Am J Clin Nutr. 2025 Oct 7;122(6):1778–87. doi: 10.1016/j.ajcnut.2025.09.051 (PMC12799371; doi:10.1016/j.ajcnut.2025.09.051)
Supplement: multimedia component 1 [file mmc1.docx]

**Supplemental Materials**

**Mediterranean diet, metabolic signature, genetic predisposition, and risk of rheumatoid arthritis: a large-scale population-based prospective cohort study**

Author list: Xin Song, Xiaofeng Ma, Bin Yang, Di Zhang, Yanqiu Zou, Bowen Lei, Rong Xiang, Xunying Zhao, Yang Qu, Sirui Zheng, Ting Yu, Jinyu Zhou, Tao Han, Yangdan Zhong, Maoyao Xia, Lars Alfredsson, Karin Leander, Mengyu Fan*, Xia Jiang*

**Table of contents**

**[Supplemental Methods](#_Toc194923891)**[................................................................................................2](#_Toc194923891)

**Supplemental Table 1**. Criteria for the MED diet score constructed to evaluate the MED diet and example of food items in the UK Biobank.............................................3

**Supplemental Table 2**. Metabolic biomarkers and abbreviations included the UK Biobank..........................................................................................................................5

**Supplemental Table 3**. Metabolites significantly associated with the MED diet score in multivariable-adjusted linear regression..................................................................19

**Supplemental Table 4**. Metabolites significantly associated with the MED diet score in elastic net regression................................................................................................27

**Supplemental Table 5**. Association between repeated assessments of metabolic signature with RA risk..................................................................................................30

**Supplemental Table 6**. Association between PRS and risk of RA.............................31

**Supplemental Table 7**. Subgroup analysis of the association between MED diet score and the risk of RA by genetic risk................................................................................32

**Supplemental Table 8**. Subgroup analysis of the association between metabolic signature and the risk of RA by genetic risk................................................................34

**Supplemental Table 9**. Interaction effects between MED diet score, metabolic signature and the risk of RA by genetic risk................................................................................................................................36

**Supplemental Table 10**. Associations between the recomputed MED diet score and risk of RA in the UK Biobank......................................................................................37

**Supplemental Table 11**. Sensitivity analyses for associations between MED diet score and risk of RA.....................................................................................................38

**Supplemental Figure 1**. Flowchart of study participant selection..............................40

**Supplemental Figure 2**. Correlation matrix for all the 251 metabolites considered in the primary analysis.....................................................................................................41

**Supplemental Figure 3**. Restricted cubic spline models for the association between (A) the MED diet score, (B) metabolic signature and risk of RA................................42

**Supplemental Figure 4**. Adjusted HR (95%CI) for MED diet score, metabolic signature (comparing the 90^th^ with the 10^th^ percentiles) and risk of RA stratified by potential risk factors.....................................................................................................43

**Supplemental Methods**

**Metabolomics measurement**

Baseline plasma samples from approximately 280,000 randomly selected UK Biobank participants were profiled using a high-throughput nuclear magnetic resonance (NMR) metabolomics platform (Nightingale Health Ltd., Helsinki, Finland). Detailed protocols for sample collection, handling, and metabolomic quantification have been reported previously ^[1,2]^. In total, 251 metabolic biomarkers were quantified, comprising 170 directly measured metabolites and 81 derived ratios, capturing a broad range of metabolic pathways. These included lipoprotein lipids across 14 subclasses, fatty acids and their compositions, as well as multiple low-molecular-weight metabolites such as amino acids, glycolysis intermediates, and ketone bodies, all quantified in molar concentration units. The lower limit of quantification for the NMR platform is approximately 10 µmol/L, although the exact threshold varies across metabolites. Concentrations below this detection limit were coded as missing, and no truncation was applied to values above the quantifiable range. To account for missing data in downstream analyses, values below the detection limit were imputed as half of the minimum observed concentration for each metabolite. Consistent with previous reports, strong correlations were observed among metabolites within the same biochemical category (**Supplemental Figure 2**).

| **Supplemental Table 1. Criteria for the MED diet score constructed to evaluate the MED diet and example of food items in the UK Biobank.** | | | | |
| --- | --- | --- | --- | --- |
| **Food component** | **Contributing foods from the Oxford WebQ** | **Unit** | **Component score** | |
|  |  |  | 0 | 1 |
| Vegetables | Mixed vegetables, vegetable pieces, coleslaw, side salad, avocado, green beans, beetroot, broccoli, butternut squash, cabbage/kale, carrot, cauliflower, celery, courgette, cucumber, garlic, leek, lettuce, mushroom, onion, parsnip, peas, sweet pepper, spinach, sprouts, sweetcorn, sweet potato, fresh tomato, tin tomato, turnip/swede, watercress, other vegetable, vegetables from canned or homemade soup, guacamole-based sauce | Serving/day | <2 | ≥2 |
| Fruit (including fruit juice) | Stewed fruit, prune, dried fruit, mixed fruit, apple, banana, berry, cherry, grapefruit, grape, mango, melon, orange, satsuma, peach/ nectarine, pear, pineapple, plum, other fruit, olives, grapefruit juice, orange juice, pure fruit/vegetable juice intake | Serving/day | <3 | ≥3 |
| Olive oil | Type of fat/ oil used for cooking | - | No | Yes |
| Red and processed meat | Beef, pork, lamb, red meat from canned or homemade soup, bacon, ham, sausage, liver | Serving/day | ≥1 | <1 |
| White meat | Poultry, breaded poultry, white meat from canned or homemade soup | - | Less white meat than red meat | More white meat than red meat |
| Butter and margarine | Butter/ margarine on potato, baguettes with butter/ margarine, baps with butter/ margarine, bread rolls with butter/ margarine, bread slices with butter/ margarine, crackers/ crispbread with butter/ margarine, oatcakes with butter/ margarine, other bread with butter/ margarine, butter/ margarine used in cooking, cream | Serving/day | ≥1 | <1 |
| Sugary drinks | Fizzy drinks, low calories drinks, squash | Serving/day | ≥1 | <1 |
| Wine | Red wine, rose wine, white wine | Serving/week | <2 | ≥2 |
| Legumes | Broad beans, baked beans, pulses, tofu, hummus-based sauce, pulses from canned or homemade soup | Serving/week | <3 | ≥3 |
| Nuts | Unsalted nuts, salted nuts, unsalted peanuts, salted peanuts, peanut butter-based sauce, seeds | Serving/week | <3 | ≥3 |
| Seafood | Battered fish, breaded fish, white fish, oily fish, shellfish, other fish, tinned tuna, prawn, lobster/crab, fish from canned or homemade soup | Serving/week | <3 | ≥3 |
| Sweets and desserts | Chocolate biscuit, chocolate covered biscuit, chocolate bar, chocolate sweets, chocolate raisins, dark chocolate, milk chocolate, white chocolate, sweet biscuits, sweets, diet sweets, other sweets, cakes, cheesecake, doughnut, fruitcake, Danish pastry, sponge pudding, milk-based pudding, other milk-based pudding, other desert, soya desert, ice cream, cereal bar, double crust pastry, single crust pastry, crumble, pancake, scotch pancake, Yorkshire pudding, croissant, scone | Serving/week | ≥2 | <2 |
| Sofrito | Tomato-based sauce | Serving/week | <2 | ≥2 |

| **Supplemental Table 2. Metabolic biomarkers and abbreviations included the UK Biobank.** | | | |
| --- | --- | --- | --- |
| Metabolite | Type | Abbreviations | Field ID |
| Glucose_lactate | Glycolysis related metabolites | Glucose_lactate | 20280 |
| Spectrometer_corrected alanine | Amino acids | Spectrometer_corrected alanine | 20281 |
| Total Cholesterol | Cholesterol | Total_C | 23400 |
| Total Cholesterol Minus HDL_C | Cholesterol | Non_HDL_C | 23401 |
| Remnant Cholesterol (Non_HDL, Non_LDL _Cholesterol) | Cholesterol | Remnant_C | 23402 |
| VLDL Cholesterol | Cholesterol | VLDL Cholesterol | 23403 |
| Clinical LDL Cholesterol | Cholesterol | Clinical_LDL_C | 23404 |
| LDL Cholesterol | Cholesterol | LDL_C | 23405 |
| HDL Cholesterol | Cholesterol | HDL_C | 23406 |
| Total Triglycerides | Triglycerides | Total_TG | 23407 |
| Triglycerides in VLDL | Triglycerides | VLDL_TG | 23408 |
| Triglycerides in LDL | Triglycerides | LDL_TG | 23409 |
| Triglycerides in HDL | Triglycerides | HDL_TG | 23410 |
| Total Phospholipids in Lipoprotein Particles | Phospholipids | Total_PL | 23411 |
| Phospholipids in VLDL | Phospholipids | VLDL_PL | 23412 |
| Phospholipids in LDL | Phospholipids | LDL_PL | 23413 |
| Phospholipids in HDL | Phospholipids | HDL_PL | 23414 |
| Total Esterified Cholesterol | Cholesteryl Esters | Total_CE | 23415 |
| Cholesteryl Esters in VLDL | Cholesteryl Esters | VLDL_CE | 23416 |
| Cholesteryl Esters in LDL | Cholesteryl Esters | LDL_CE | 23417 |
| Cholesteryl Esters in HDL | Cholesteryl Esters | HDL_CE | 23418 |
| Total Free Cholesterol | Free Cholesterol | Total_FC | 23419 |
| Free Cholesterol in VLDL | Free Cholesterol | VLDL_FC | 23420 |
| Free Cholesterol in LDL | Free Cholesterol | LDL_FC | 23421 |
| Free Cholesterol in HDL | Free Cholesterol | HDL_FC | 23422 |
| Total Lipids in Lipoprotein Particles | Total Lipids | Total_L | 23423 |
| Total Lipids in VLDL | Total Lipids | VLDL_L | 23424 |
| Total Lipids in LDL | Total Lipids | LDL_L | 23425 |
| Total Lipids in HDL | Total Lipids | HDL_L | 23426 |
| Total Concentration of Lipoprotein Particles | Lipoprotein Particle Concentrations | Total_P | 23427 |
| Concentration of VLDL Particles | Lipoprotein Particle Concentrations | VLDL_P | 23428 |
| Concentration of LDL Particles | Lipoprotein Particle Concentrations | LDL_P | 23429 |
| Concentration of HDL Particles | Lipoprotein Particle Concentrations | HDL_P | 23430 |
| Average Diameter for VLDL Particles | Lipoprotein Particle Size | VLDL_size | 23431 |
| Average Diameter for LDL Particles | Lipoprotein Particle Size | LDL_size | 23432 |
| Average Diameter for HDL Particles | Lipoprotein Particle Size | HDL_size | 23433 |
| Phosphoglycerides | Other lipids | Phosphoglycerides | 23434 |
| Ratio of triglycerides to phosphoglycerides | Other lipids | TGvsPG_ratio | 23435 |
| Total Cholines | Other lipids | Cholines | 23436 |
| Phosphatidylcholines | Other lipids | Phosphatidylc | 23437 |
| Sphingomyelins | Other lipids | Sphingomyelins | 23438 |
| Apolipoprotein B | Apolipoproteins | ApoB | 23439 |
| Apolipoprotein A1 | Apolipoproteins | ApoA1 | 23440 |
| Ratio of apolipoprotein B to apolipoprotein A1 | Apolipoproteins | ApoBvsApoA1_ratio | 23441 |
| Total Fatty Acids | Fatty Acids | Total_FA | 23442 |
| Degree of Unsaturation | Fatty Acids | Unsaturation | 23443 |
| Omega_3 Fatty Acids | Fatty Acids | Omega_3 | 23444 |
| Omega_6 Fatty Acids | Fatty Acids | Omega_6 | 23445 |
| Polyunsaturated Fatty Acids | Fatty Acids | PUFA | 23446 |
| Monounsaturated Fatty Acids | Fatty Acids | MUFA | 23447 |
| Saturated Fatty Acids | Fatty Acids | SFA | 23448 |
| Linoleic Acid | Fatty Acids | LA | 23449 |
| Docosahexaenoic Acid | Fatty Acids | DHA | 23450 |
| Ratio of omega_3 fatty acids to total fatty acids | Fatty acids | ω_3_Total_FA_ratio | 23451 |
| Ratio of omega_6 fatty acids to total fatty acids | Fatty acids | ω_6_Total_FA_ratio | 23452 |
| Ratio of polyunsaturated fatty acids to total fatty acids | Fatty acids | PUFA_Total_FA_ratio | 23453 |
| Ratio of monounsaturated fatty acids to total fatty acids | Fatty acids | MUFA_Total_FA_ratio | 23454 |
| Ratio of saturated fatty acids to total fatty acids | Fatty acids | SFA_Total_FA_ratio | 23455 |
| Ratio of linoleic acid to total fatty acids | Fatty acids | LA_Total_FA_ratio | 23456 |
| Ratio of docosahexaenoic acid to total fatty acids | Fatty acids | DHA_Total_FA_ratio | 23457 |
| Ratio of polyunsaturated fatty acids to monounsaturated fatty acids | Fatty acids | PUFA_MUFA_ratio | 23458 |
| Ratio of omega_6 fatty acids to omega_3 fatty acids | Fatty acids | ω_6_ω_3_ratio | 23459 |
| Alanine | Amino acids | Alanine | 23460 |
| Glutamine | Amino acids | Glutamine | 23461 |
| Glycine | Amino acids | Glycine | 23462 |
| Histidine | Amino acids | Histidine | 23463 |
| Total Concentration of Branched_Chain Amino Acids (Leucine + Isoleucine + Valine) | Amino acids | Total_BCAA | 23464 |
| Isoleucine | Amino acids | Isoleucine | 23465 |
| Leucine | Amino acids | Leucine | 23466 |
| Valine | Amino acids | Valine | 23467 |
| Phenylalanine | Amino acids | Phenylalanine | 23468 |
| Tyrosine | Amino acids | Tyrosine | 23469 |
| Glucose | Glycolysis related metabolites | Glucose | 23470 |
| Lactate | Glycolysis related metabolites | Lactate | 23471 |
| Pyruvate | Glycolysis related metabolites | Pyruvate | 23472 |
| Citrate | Glycolysis related metabolites | Citrate | 23473 |
| 3_Hydroxybutyrate | Ketone bodies | bOHbutyrate | 23474 |
| Acetate | Ketone bodies | Acetate | 23475 |
| Acetoacetate | Ketone bodies | Acetoacetate | 23476 |
| Acetone | Ketone bodies | Acetone | 23477 |
| Creatinine | Fluid balance | Creatinine | 23478 |
| Albumin | Fluid balance | Albumin | 23479 |
| Glycoprotein Acetyls | Fluid balance | GlycA | 23480 |
| Concentration of Chylomicrons and Extremely Large VLDL Particles | Lipoprotein subclasses | XXL_VLDL_P | 23481 |
| Total Lipids in Chylomicrons and Extremely Large VLDL | Lipoprotein subclasses | XXL_VLDL_L | 23482 |
| Phospholipids in Chylomicrons and Extremely Large VLDL | Lipoprotein subclasses | XXL_VLDL_PL | 23483 |
| Cholesterol in Chylomicrons and Extremely Large VLDL | Lipoprotein subclasses | XXL_VLDL_C | 23484 |
| Cholesteryl Esters in Chylomicrons and Extremely Large VLDL | Lipoprotein subclasses | XXL_VLDL_CE | 23485 |
| Free Cholesterol in Chylomicrons and Extremely Large VLDL | Lipoprotein subclasses | XXL_VLDL_FC | 23486 |
| Triglycerides in Chylomicrons and Extremely Large VLDL | Lipoprotein subclasses | XXL_VLDL_TG | 23487 |
| Concentration of Very Large VLDL Particles | Lipoprotein subclasses | XL_VLDL_P | 23488 |
| Total Lipids in Very Large VLDL | Lipoprotein subclasses | XL_VLDL_L | 23489 |
| Phospholipids in Very Large VLDL | Lipoprotein subclasses | XL_VLDL_PL | 23490 |
| Cholesterol in Very Large VLDL | Lipoprotein subclasses | XL_VLDL_C | 23491 |
| Cholesteryl Esters in Very Large VLDL | Lipoprotein subclasses | XL_VLDL_CE | 23492 |
| Free Cholesterol in Very Large VLDL | Lipoprotein subclasses | XL_VLDL_FC | 23493 |
| Triglycerides in Very Large VLDL | Lipoprotein subclasses | XL_VLDL_TG | 23494 |
| Concentration of Large VLDL Particles | Lipoprotein subclasses | L_VLDL_P | 23495 |
| Total Lipids in Large VLDL | Lipoprotein subclasses | L_VLDL_L | 23496 |
| Phospholipids in Large VLDL | Lipoprotein subclasses | L_VLDL_PL | 23497 |
| Cholesterol in Large VLDL | Lipoprotein subclasses | L_VLDL_C | 23498 |
| Cholesteryl Esters in Large VLDL | Lipoprotein subclasses | L_VLDL_CE | 23499 |
| Free Cholesterol in Large VLDL | Lipoprotein subclasses | L_VLDL_FC | 23500 |
| Triglycerides in Large VLDL | Lipoprotein subclasses | L_VLDL_TG | 23501 |
| Concentration of Medium VLDL Particles | Lipoprotein subclasses | M_VLDL_P | 23502 |
| Total Lipids in Medium VLDL | Lipoprotein subclasses | M_VLDL_L | 23503 |
| Phospholipids in Medium VLDL | Lipoprotein subclasses | M_VLDL_PL | 23504 |
| Cholesterol in Medium VLDL | Lipoprotein subclasses | M_VLDL_C | 23505 |
| Cholesteryl Esters in Medium VLDL | Lipoprotein subclasses | M_VLDL_CE | 23506 |
| Free Cholesterol in Medium VLDL | Lipoprotein subclasses | M_VLDL_FC | 23507 |
| Triglycerides in Medium VLDL | Lipoprotein subclasses | M_VLDL_TG | 23508 |
| Concentration of Small VLDL Particles | Lipoprotein subclasses | S_VLDL_P | 23509 |
| Total Lipids in Small VLDL | Lipoprotein subclasses | S_VLDL_L | 23510 |
| Phospholipids in Small VLDL | Lipoprotein subclasses | S_VLDL_PL | 23511 |
| Cholesterol in Small VLDL | Lipoprotein subclasses | S_VLDL_C | 23512 |
| Cholesteryl Esters in Small VLDL | Lipoprotein subclasses | S_VLDL_CE | 23513 |
| Free Cholesterol in Small VLDL | Lipoprotein subclasses | S_VLDL_FC | 23514 |
| Triglycerides in Small VLDL | Lipoprotein subclasses | S_VLDL_TG | 23515 |
| Concentration of Very Small VLDL Particles | Lipoprotein subclasses | XS_VLDL_P | 23516 |
| Total Lipids in Very Small VLDL | Lipoprotein subclasses | XS_VLDL_L | 23517 |
| Phospholipids in Very Small VLDL | Lipoprotein subclasses | XS_VLDL_PL | 23518 |
| Cholesterol in Very Small VLDL | Lipoprotein subclasses | XS_VLDL_C | 23519 |
| Cholesteryl Esters in Very Small VLDL | Lipoprotein subclasses | XS_VLDL_CE | 23520 |
| Free Cholesterol in Very Small VLDL | Lipoprotein subclasses | XS_VLDL_FC | 23521 |
| Triglycerides in Very Small VLDL | Lipoprotein subclasses | XS_VLDL_TG | 23522 |
| Concentration of IDL Particles | Lipoprotein subclasses | IDL_P | 23523 |
| Total Lipids in IDL | Lipoprotein subclasses | IDL_L | 23524 |
| Phospholipids in IDL | Lipoprotein subclasses | IDL_PL | 23525 |
| Cholesterol in IDL | Lipoprotein subclasses | IDL_C | 23526 |
| Cholesteryl Esters in IDL | Lipoprotein subclasses | IDL_CE | 23527 |
| Free Cholesterol in IDL | Lipoprotein subclasses | IDL_FC | 23528 |
| Triglycerides in IDL | Lipoprotein subclasses | IDL_TG | 23529 |
| Concentration of Large LDL Particles | Lipoprotein subclasses | L_LDL_P | 23530 |
| Total Lipids in Large LDL | Lipoprotein subclasses | L_LDL_L | 23531 |
| Phospholipids in Large LDL | Lipoprotein subclasses | L_LDL_PL | 23532 |
| Cholesterol in Large LDL | Lipoprotein subclasses | L_LDL_C | 23533 |
| Cholesteryl Esters in Large LDL | Lipoprotein subclasses | L_LDL_CE | 23534 |
| Free Cholesterol in Large LDL | Lipoprotein subclasses | L_LDL_FC | 23535 |
| Triglycerides in Large LDL | Lipoprotein subclasses | L_LDL_TG | 23536 |
| Concentration of Medium LDL Particles | Lipoprotein subclasses | M_LDL_P | 23537 |
| Total Lipids in Medium LDL | Lipoprotein subclasses | M_LDL_L | 23538 |
| Phospholipids in Medium LDL | Lipoprotein subclasses | M_LDL_PL | 23539 |
| Cholesterol in Medium LDL | Lipoprotein subclasses | M_LDL_C | 23540 |
| Cholesteryl Esters in Medium LDL | Lipoprotein subclasses | M_LDL_CE | 23541 |
| Free Cholesterol in Medium LDL | Lipoprotein subclasses | M_LDL_FC | 23542 |
| Triglycerides in Medium LDL | Lipoprotein subclasses | M_LDL_TG | 23543 |
| Concentration of Small LDL Particles | Lipoprotein subclasses | S_LDL_P | 23544 |
| Total Lipids in Small LDL | Lipoprotein subclasses | S_LDL_L | 23545 |
| Phospholipids in Small LDL | Lipoprotein subclasses | S_LDL_PL | 23546 |
| Cholesterol in Small LDL | Lipoprotein subclasses | S_LDL_C | 23547 |
| Cholesteryl Esters in Small LDL | Lipoprotein subclasses | S_LDL_CE | 23548 |
| Free Cholesterol in Small LDL | Lipoprotein subclasses | S_LDL_FC | 23549 |
| Triglycerides in Small LDL | Lipoprotein subclasses | S_LDL_TG | 23550 |
| Concentration of Very Large HDL Particles | Lipoprotein subclasses | XL_HDL_P | 23551 |
| Total Lipids in Very Large HDL | Lipoprotein subclasses | XL_HDL_L | 23552 |
| Phospholipids in Very Large HDL | Lipoprotein subclasses | XL_HDL_PL | 23553 |
| Cholesterol in Very Large HDL | Lipoprotein subclasses | XL_HDL_C | 23554 |
| Cholesteryl Esters in Very Large HDL | Lipoprotein subclasses | XL_HDL_CE | 23555 |
| Free Cholesterol in Very Large HDL | Lipoprotein subclasses | XL_HDL_FC | 23556 |
| Triglycerides in Very Large HDL | Lipoprotein subclasses | XL_HDL_TG | 23557 |
| Concentration of Large HDL Particles | Lipoprotein subclasses | L_HDL_P | 23558 |
| Total Lipids in Large HDL | Lipoprotein subclasses | L_HDL_L | 23559 |
| Phospholipids in Large HDL | Lipoprotein subclasses | L_HDL_PL | 23560 |
| Cholesterol in Large HDL | Lipoprotein subclasses | L_HDL_C | 23561 |
| Cholesteryl Esters in Large HDL | Lipoprotein subclasses | L_HDL_CE | 23562 |
| Free Cholesterol in Large HDL | Lipoprotein subclasses | L_HDL_FC | 23563 |
| Triglycerides in Large HDL | Lipoprotein subclasses | L_HDL_TG | 23564 |
| Concentration of Medium HDL Particles | Lipoprotein subclasses | M_HDL_P | 23565 |
| Total Lipids in Medium HDL | Lipoprotein subclasses | M_HDL_L | 23566 |
| Phospholipids in Medium HDL | Lipoprotein subclasses | M_HDL_PL | 23567 |
| Cholesterol in Medium HDL | Lipoprotein subclasses | M_HDL_C | 23568 |
| Cholesteryl Esters in Medium HDL | Lipoprotein subclasses | M_HDL_CE | 23569 |
| Free Cholesterol in Medium HDL | Lipoprotein subclasses | M_HDL_FC | 23570 |
| Triglycerides in Medium HDL | Lipoprotein subclasses | M_HDL_TG | 23571 |
| Concentration of Small HDL Particles | Lipoprotein subclasses | S_HDL_P | 23572 |
| Total Lipids in Small HDL | Lipoprotein subclasses | S_HDL_L | 23573 |
| Phospholipids in Small HDL | Lipoprotein subclasses | S_HDL_PL | 23574 |
| Cholesterol in Small HDL | Lipoprotein subclasses | S_HDL_C | 23575 |
| Cholesteryl Esters in Small HDL | Lipoprotein subclasses | S_HDL_CE | 23576 |
| Free Cholesterol in Small HDL | Lipoprotein subclasses | S_HDL_FC | 23577 |
| Triglycerides in Small HDL | Lipoprotein subclasses | S_HDL_TG | 23578 |
| Phospholipids to Total Lipids in Chylomicrons and Extremely Large VLDL percentage | Relative lipoprotein lipid concentrations | PL_TL in CM & ELVLDL_perce | 23579 |
| Cholesterol to Total Lipids in Chylomicrons and Extremely Large VLDL percentage | Relative lipoprotein lipid concentrations | C_TL in CM & ELVDL_perce | 23580 |
| Cholesteryl Esters to Total Lipids in Chylomicrons and Extremely Large VLDL percentage | Relative lipoprotein lipid concentrations | CE_TL in CM & ELVDL_perce | 23581 |
| Free Cholesterol to Total Lipids in Chylomicrons and Extremely Large VLDL percentage | Relative lipoprotein lipid concentrations | FC_TL in CM & ELVDL_perce | 23582 |
| Triglycerides to Total Lipids in Chylomicrons and Extremely Large VLDL percentage | Relative lipoprotein lipid concentrations | TG_TL in CM & ELVDL_perce | 23583 |
| Phospholipids to Total Lipids in Very Large VLDL percentage | Relative lipoprotein lipid concentrations | PL_TL in VLVDL_perce | 23584 |
| Cholesterol to Total Lipids in Very Large VLDL percentage | Relative lipoprotein lipid concentrations | C_TL in VLVDL_perce | 23585 |
| Cholesteryl Esters to Total Lipids in Very Large VLDL percentage | Relative lipoprotein lipid concentrations | CE_TL in VLVDL_perce | 23586 |
| Free Cholesterol to Total Lipids in Very Large VLDL percentage | Relative lipoprotein lipid concentrations | FC_TL in VLVDL_perce | 23587 |
| Triglycerides to Total Lipids in Very Large VLDL percentage | Relative lipoprotein lipid concentrations | TG_TL in VLVDL_perce | 23588 |
| Phospholipids to Total Lipids in Large VLDL percentage | Relative lipoprotein lipid concentrations | PL_TL in LVDL_perce | 23589 |
| Cholesterol to Total Lipids in Large VLDL percentage | Relative lipoprotein lipid concentrations | C_TL in LVDL_perce | 23590 |
| Cholesteryl Esters to Total Lipids in Large VLDL percentage | Relative lipoprotein lipid concentrations | CE_TL in LVDL_perce | 23591 |
| Free Cholesterol to Total Lipids in Large VLDL percentage | Relative lipoprotein lipid concentrations | FC_TL in LVDL_perce | 23592 |
| Triglycerides to Total Lipids in Large VLDL percentage | Relative lipoprotein lipid concentrations | TG_TL in LVDL_perce | 23593 |
| Phospholipids to Total Lipids in Medium VLDL percentage | Relative lipoprotein lipid concentrations | PL_TL in MVLDL_perce | 23594 |
| Cholesterol to Total Lipids in Medium VLDL percentage | Relative lipoprotein lipid concentrations | C_TL in MVLDL_perce | 23595 |
| Cholesteryl Esters to Total Lipids in Medium VLDL percentage | Relative lipoprotein lipid concentrations | CE_TL in MVLDL_perce | 23596 |
| Free Cholesterol to Total Lipids in Medium VLDL percentage | Relative lipoprotein lipid concentrations | FE_TL in MVLDL_perce | 23597 |
| Triglycerides to Total Lipids in Medium VLDL percentage | Relative lipoprotein lipid concentrations | TG_TL in MVLDL_perce | 23598 |
| Phospholipids to Total Lipids in Small VLDL percentage | Relative lipoprotein lipid concentrations | PL_TL in MVLDL_perce | 23599 |
| Cholesterol to Total Lipids in Small VLDL percentage | Relative lipoprotein lipid concentrations | C_TL in SVLDL_perce | 23600 |
| Cholesteryl Esters to Total Lipids in Small VLDL percentage | Relative lipoprotein lipid concentrations | CE_TL in SVLDL_perce | 23601 |
| Free Cholesterol to Total Lipids in Small VLDL percentage | Relative lipoprotein lipid concentrations | FC_TL in SVLDL_perce | 23602 |
| Triglycerides to Total Lipids in Small VLDL percentage | Relative lipoprotein lipid concentrations | TG_TL in SVLDL_perce | 23603 |
| Phospholipids to Total Lipids in Very Small VLDL percentage | Relative lipoprotein lipid concentrations | PL_TL in VSVLDL_perce | 23604 |
| Cholesterol to Total Lipids in Very Small VLDL percentage | Relative lipoprotein lipid concentrations | C_TL in VSVLDL_perce | 23605 |
| Cholesteryl Esters to Total Lipids in Very Small VLDL percentage | Relative lipoprotein lipid concentrations | CE_TL in VSVLDL_perce | 23606 |
| Free Cholesterol to Total Lipids in Very Small VLDL percentage | Relative lipoprotein lipid concentrations | FC_TL in VSVLDL_perce | 23607 |
| Triglycerides to Total Lipids in Very Small VLDL percentage | Relative lipoprotein lipid concentrations | TG_TL in VSVLDL_perce | 23608 |
| Phospholipids to Total Lipids in IDL percentage | Relative lipoprotein lipid concentrations | PL_TL in IDL_perce | 23609 |
| Cholesterol to Total Lipids in IDL percentage | Relative lipoprotein lipid concentrations | C_TL in IDL_perce | 23610 |
| Cholesteryl Esters to Total Lipids in IDL percentage | Relative lipoprotein lipid concentrations | CE_TL in IDL_perce | 23611 |
| Free Cholesterol to Total Lipids in IDL percentage | Relative lipoprotein lipid concentrations | FC_TL in IDL_perce | 23612 |
| Triglycerides to Total Lipids in IDL percentage | Relative lipoprotein lipid concentrations | TG_TL in IDL_perce | 23613 |
| Phospholipids to Total Lipids in Large LDL percentage | Relative lipoprotein lipid concentrations | PL_TL in LLDL_perce | 23614 |
| Cholesterol to Total Lipids in Large LDL percentage | Relative lipoprotein lipid concentrations | C_TL in LLDL_perce | 23615 |
| Cholesteryl Esters to Total Lipids in Large LDL percentage | Relative lipoprotein lipid concentrations | CE_TL in LLDL_perce | 23616 |
| Free Cholesterol to Total Lipids in Large LDL percentage | Relative lipoprotein lipid concentrations | FC_TL in LLDL_perce | 23617 |
| Triglycerides to Total Lipids in Large LDL percentage | Relative lipoprotein lipid concentrations | TG_TL in LLDL_perce | 23618 |
| Phospholipids to Total Lipids in Medium LDL percentage | Relative lipoprotein lipid concentrations | PL_TL in MLDL_perce | 23619 |
| Cholesterol to Total Lipids in Medium LDL percentage | Relative lipoprotein lipid concentrations | C_TL in MLDL_perce | 23620 |
| Cholesteryl Esters to Total Lipids in Medium LDL percentage | Relative lipoprotein lipid concentrations | CE_TL in MLDL_perce | 23621 |
| Free Cholesterol to Total Lipids in Medium LDL percentage | Relative lipoprotein lipid concentrations | FC_TL in MLDL_perce | 23622 |
| Triglycerides to Total Lipids in Medium LDL percentage | Relative lipoprotein lipid concentrations | TG_TL in MLDL_perce | 23623 |
| Phospholipids to Total Lipids in Small LDL percentage | Relative lipoprotein lipid concentrations | PL_TL in SLDL_perce | 23624 |
| Cholesterol to Total Lipids in Small LDL percentage | Relative lipoprotein lipid concentrations | C_TL in SLDL_perce | 23625 |
| Cholesteryl Esters to Total Lipids in Small LDL percentage | Relative lipoprotein lipid concentrations | CE_TL in SLDL_perce | 23626 |
| Free Cholesterol to Total Lipids in Small LDL percentage | Relative lipoprotein lipid concentrations | FC_TL in SLDL_perce | 23627 |
| Triglycerides to Total Lipids in Small LDL percentage | Relative lipoprotein lipid concentrations | TG_TL in SLDL_perce | 23628 |
| Phospholipids to Total Lipids in Very Large HDL percentage | Relative lipoprotein lipid concentrations | PL_TL in VLHDL_perce | 23629 |
| Cholesterol to Total Lipids in Very Large HDL percentage | Relative lipoprotein lipid concentrations | C_TL in VLHDL_perce | 23630 |
| Cholesteryl Esters to Total Lipids in Very Large HDL percentage | Relative lipoprotein lipid concentrations | CE_TL in VLHDL_perce | 23631 |
| Free Cholesterol to Total Lipids in Very Large HDL percentage | Relative lipoprotein lipid concentrations | FC_TL in VLHDL_perce | 23632 |
| Triglycerides to Total Lipids in Very Large HDL percentage | Relative lipoprotein lipid concentrations | TG_TL in VLHDL_perce | 23633 |
| Phospholipids to Total Lipids in Large HDL percentage | Relative lipoprotein lipid concentrations | PL_TL in LHDL_perce | 23634 |
| Cholesterol to Total Lipids in Large HDL percentage | Relative lipoprotein lipid concentrations | C_TL in LHDL_perce | 23635 |
| Cholesteryl Esters to Total Lipids in Large HDL percentage | Relative lipoprotein lipid concentrations | CE_TL in LHDL_perce | 23636 |
| Free Cholesterol to Total Lipids in Large HDL percentage | Relative lipoprotein lipid concentrations | FC_TL in LHDL_perce | 23637 |
| Triglycerides to Total Lipids in Large HDL percentage | Relative lipoprotein lipid concentrations | TG_TL in LHDL_perce | 23638 |
| Phospholipids to Total Lipids in Medium HDL percentage | Relative lipoprotein lipid concentrations | PL_TL in MHDL_perce | 23639 |
| Cholesterol to Total Lipids in Medium HDL percentage | Relative lipoprotein lipid concentrations | C_TL in MHDL_perce | 23640 |
| Cholesteryl Esters to Total Lipids in Medium HDL percentage | Relative lipoprotein lipid concentrations | CE_TL in MHDL_perce | 23641 |
| Free Cholesterol to Total Lipids in Medium HDL percentage | Relative lipoprotein lipid concentrations | FC_TL in MHDL_perce | 23642 |
| Triglycerides to Total Lipids in Small HDL percentage | Relative lipoprotein lipid concentrations | TG_TL in SHDL_perce | 23643 |
| Phospholipids to Total Lipids in Small HDL percentage | Relative lipoprotein lipid concentrations | PL_TL in SHDL_perce | 23644 |
| Cholesterol to Total Lipids in Small HDL percentage | Relative lipoprotein lipid concentrations | C_TL in SHDL_perce | 23645 |
| Cholesteryl Esters to Total Lipids in Small HDL percentage | Relative lipoprotein lipid concentrations | CE_TL in SHDL_perce | 23646 |
| Free Cholesterol to Total Lipids in Small HDL percentage | Relative lipoprotein lipid concentrations | FC_TL in SHDL_perce | 23647 |
| Triglycerides to Total Lipids in Small HDL percentage | Relative lipoprotein lipid concentrations | TG_TL in SHDL_perce | 23648 |

| **Supplemental Table 3. Metabolites significantly associated with the MED diet score in multivariable-adjusted linear regression.** | | | |
| --- | --- | --- | --- |
| **Metabolites** | **Type** | **Beta^1^** | ***P^2^*** |
| Spectrometer_corrected alanine | Amino acids | -0.022466552 | 1.59557E-05 |
| Total_C | Cholesterol | 0.042742809 | 2.20061E-15 |
| VLDL Cholesterol | Cholesterol | -0.053729757 | 4.74431E-25 |
| HDL_C | Cholesterol | 0.15280916 | 7.289E-147 |
| Total_TG | Triglycerides | -0.09099767 | 1.99211E-61 |
| VLDL_TG | Triglycerides | -0.100642315 | 1.69982E-73 |
| LDL_TG | Triglycerides | -0.024153913 | 6.72554E-06 |
| HDL_TG | Triglycerides | -0.053407192 | 7.655E-24 |
| Total_PL | Phospholipids | 0.050148117 | 1.70784E-20 |
| VLDL_PL | Phospholipids | -0.072984966 | 8.74502E-43 |
| HDL_PL | Phospholipids | 0.118761516 | 6.05736E-96 |
| Total_CE | Cholesteryl Esters | 0.050174864 | 1.90855E-20 |
| VLDL_CE | Cholesteryl Esters | -0.043740541 | 2.7688E-17 |
| HDL_CE | Cholesteryl Esters | 0.153132821 | 3.5275E-148 |
| Total_FC | Free Cholesterol | 0.023310203 | 1.12464E-05 |
| VLDL_FC | Free Cholesterol | -0.063992203 | 6.79715E-34 |
| LDL_FC | Free Cholesterol | 0.025300774 | 1.5025E-06 |
| HDL_FC | Free Cholesterol | 0.141750795 | 8.3144E-130 |
| VLDL_L | Total Lipids | -0.087308791 | 3.64308E-59 |
| HDL_L | Total Lipids | 0.129794424 | 4.2091E-111 |
| Total_P | Lipoprotein Particle Concentrations | 0.090101168 | 7.48455E-62 |
| VLDL_P | Lipoprotein Particle Concentrations | -0.05092294 | 2.58342E-22 |
| HDL_P | Lipoprotein Particle Concentrations | 0.096968397 | 5.11622E-71 |
| VLDL_size | Lipoprotein Particle Concentrations | -0.135905725 | 8.0318E-124 |
| LDL_size | Lipoprotein Particle Concentrations | 0.083511277 | 4.65319E-55 |
| HDL_size | Lipoprotein Particle Concentrations | 0.135745645 | 4.4229E-114 |
| Phosphoglycerides | Other lipids | 0.064558839 | 1.9591E-33 |
| TGvsPG_ratio | Other lipids | -0.142136426 | 2.6778E-130 |
| Cholines | Other lipids | 0.067384715 | 1.36495E-35 |
| Phosphatidylc | Other lipids | 0.071588216 | 8.51141E-40 |
| Sphingomyelins | Other lipids | 0.071398902 | 7.91888E-39 |
| ApoA1 | Apolipoproteins | 0.112138204 | 5.43889E-88 |
| ApoBvsApoA1_ratio | Apolipoproteins | -0.068665178 | 1.95193E-38 |
| Unsaturation | Fatty Acids | 0.257915881 | 0 |
| Omega_3 | Fatty Acids | 0.21548908 | 0 |
| PUFA | Fatty Acids | 0.066596227 | 9.78975E-38 |
| MUFA | Fatty Acids | -0.067824548 | 9.9021E-37 |
| SFA | Fatty Acids | -0.028789067 | 3.89486E-08 |
| DHA | Fatty Acids | 0.272769181 | 0 |
| ω_3_Total_FA_ratio | Fatty Acids | 0.264349345 | 0 |
| ω_6_Total_FA_ratio | Fatty Acids | 0.036153376 | 1.20124E-10 |
| PUFA_Total_FA_ratio | Fatty Acids | 0.164522274 | 1.0831E-181 |
| MUFA_Total_FA_ratio | Fatty Acids | -0.14083924 | 1.4304E-164 |
| SFA_Total_FA_ratio | Fatty Acids | -0.040466316 | 8.52807E-28 |
| DHA_Total_FA_ratio | Fatty Acids | 0.298024457 | 0 |
| PUFA_MUFA_ratio | Fatty Acids | 0.187452556 | 8.1884E-229 |
| ω_6_ω_3_ratio | Fatty Acids | -0.228195215 | 0 |
| Glycine | Amino acids | -0.022278716 | 3.35031E-05 |
| Isoleucine | Amino acids | -0.026521632 | 3.94914E-07 |
| Leucine | Amino acids | -0.026011745 | 1.16162E-06 |
| Phenylalanine | Amino acids | -0.026799197 | 4.29114E-07 |
| Tyrosine | Amino acids | 0.024083722 | 4.14598E-06 |
| Lactate | Glycolysis related metabolites | -0.027875672 | 1.36491E-08 |
| Citrate | Glycolysis related metabolites | -0.051572172 | 9.50205E-24 |
| bOHbutyrate | Ketone bodies | 0.05004325 | 1.19967E-21 |
| Acetate | Ketone bodies | 0.044079945 | 3.36763E-13 |
| Acetoacetate | Ketone bodies | 0.047936237 | 7.25957E-20 |
| Acetone | Ketone bodies | 0.065251979 | 9.15035E-37 |
| Creatinine | Fluid balance | -0.115567049 | 2.87308E-90 |
| Albumin | Fluid balance | 0.076214355 | 2.00213E-55 |
| GlycA | Fluid balance | -0.099267383 | 3.75872E-73 |
| XXL_VLDL_P | Lipoprotein subclasses | -0.094506933 | 6.6756E-63 |
| XXL_VLDL_L | Lipoprotein subclasses | -0.104647801 | 2.86737E-77 |
| XXL_VLDL_PL | Lipoprotein subclasses | -0.095742034 | 1.67313E-64 |
| XXL_VLDL_C | Lipoprotein subclasses | -0.100576211 | 1.36577E-72 |
| XXL_VLDL_CE | Lipoprotein subclasses | -0.104521511 | 6.7441E-79 |
| XXL_VLDL_FC | Lipoprotein subclasses | -0.091786189 | 2.68168E-60 |
| XXL_VLDL_TG | Lipoprotein subclasses | -0.101322085 | 2.04917E-72 |
| XL_VLDL_P | Lipoprotein subclasses | -0.099717444 | 7.71252E-71 |
| XL_VLDL_L | Lipoprotein subclasses | -0.106131017 | 3.14187E-80 |
| XL_VLDL_PL | Lipoprotein subclasses | -0.100595961 | 6.81737E-73 |
| XL_VLDL_C | Lipoprotein subclasses | -0.095014411 | 2.53859E-68 |
| XL_VLDL_CE | Lipoprotein subclasses | -0.089700947 | 4.83955E-63 |
| XL_VLDL_FC | Lipoprotein subclasses | -0.096186536 | 7.60092E-68 |
| XL_VLDL_TG | Lipoprotein subclasses | -0.104070653 | 7.52558E-76 |
| L_VLDL_P | Lipoprotein subclasses | -0.093288893 | 4.48762E-64 |
| L_VLDL_L | Lipoprotein subclasses | -0.100543677 | 1.13849E-74 |
| L_VLDL_PL | Lipoprotein subclasses | -0.10073176 | 5.66777E-74 |
| L_VLDL_C | Lipoprotein subclasses | -0.088402843 | 1.64192E-60 |
| L_VLDL_CE | Lipoprotein subclasses | -0.078031198 | 1.69374E-49 |
| L_VLDL_FC | Lipoprotein subclasses | -0.095134208 | 7.69126E-67 |
| L_VLDL_TG | Lipoprotein subclasses | -0.096556072 | 1.99281E-68 |
| M_VLDL_P | Lipoprotein subclasses | -0.047890012 | 2.97902E-20 |
| M_VLDL_L | Lipoprotein subclasses | -0.060759277 | 3.42436E-31 |
| M_VLDL_PL | Lipoprotein subclasses | -0.045848395 | 9.31685E-19 |
| M_VLDL_FC | Lipoprotein subclasses | -0.034701507 | 1.80369E-11 |
| M_VLDL_TG | Lipoprotein subclasses | -0.083066783 | 5.04782E-53 |
| S_VLDL_P | Lipoprotein subclasses | -0.054707003 | 3.67369E-25 |
| S_VLDL_L | Lipoprotein subclasses | -0.055126044 | 9.39203E-26 |
| S_VLDL_PL | Lipoprotein subclasses | -0.03788512 | 2.5881E-13 |
| S_VLDL_C | Lipoprotein subclasses | -0.037885385 | 2.15179E-13 |
| S_VLDL_CE | Lipoprotein subclasses | -0.04410232 | 1.5757E-17 |
| S_VLDL_FC | Lipoprotein subclasses | -0.025379787 | 8.46134E-07 |
| S_VLDL_TG | Lipoprotein subclasses | -0.066619716 | 1.57915E-34 |
| XS_VLDL_TG | Lipoprotein subclasses | -0.03615436 | 1.68003E-11 |
| IDL_L | Lipoprotein subclasses | 0.032323481 | 2.12096E-09 |
| IDL_PL | Lipoprotein subclasses | 0.030613829 | 1.26462E-08 |
| IDL_C | Lipoprotein subclasses | 0.036421022 | 2.10073E-11 |
| IDL_CE | Lipoprotein subclasses | 0.033552385 | 6.27693E-10 |
| IDL_FC | Lipoprotein subclasses | 0.042111369 | 6.73939E-15 |
| L_LDL_L | Lipoprotein subclasses | 0.0233351 | 8.55877E-06 |
| L_LDL_C | Lipoprotein subclasses | 0.019643296 | 0.000162939 |
| L_LDL_CE | Lipoprotein subclasses | 0.032065278 | 1.54172E-09 |
| L_LDL_FC | Lipoprotein subclasses | -0.029052479 | 1.79353E-08 |
| M_LDL_P | Lipoprotein subclasses | -0.025218114 | 1.07427E-06 |
| M_LDL_CE | Lipoprotein subclasses | -0.03272817 | 1.53893E-09 |
| M_LDL_TG | Lipoprotein subclasses | -0.029049527 | 1.97349E-08 |
| S_LDL_P | Lipoprotein subclasses | -0.024330623 | 2.66612E-06 |
| S_LDL_CE | Lipoprotein subclasses | -0.060485062 | 4.1709E-28 |
| S_LDL_TG | Lipoprotein subclasses | 0.11640472 | 8.83714E-91 |
| XL_HDL_P | Lipoprotein subclasses | 0.125296486 | 5.3327E-102 |
| XL_HDL_L | Lipoprotein subclasses | 0.122032352 | 4.50364E-98 |
| XL_HDL_PL | Lipoprotein subclasses | 0.130422902 | 4.0069E-110 |
| XL_HDL_C | Lipoprotein subclasses | 0.138625903 | 1.9895E-122 |
| XL_HDL_CE | Lipoprotein subclasses | 0.093151997 | 5.22262E-62 |
| XL_HDL_FC | Lipoprotein subclasses | 0.149441625 | 6.9754E-139 |
| L_HDL_P | Lipoprotein subclasses | 0.156865955 | 5.1259E-147 |
| L_HDL_L | Lipoprotein subclasses | 0.152534249 | 1.3522E-143 |
| L_HDL_PL | Lipoprotein subclasses | 0.160040679 | 5.8345E-153 |
| L_HDL_C | Lipoprotein subclasses | 0.160322847 | 4.9069E-154 |
| L_HDL_CE | Lipoprotein subclasses | 0.149855823 | 1.6944E-139 |
| L_HDL_FC | Lipoprotein subclasses | 0.110210969 | 5.78591E-87 |
| M_HDL_P | Lipoprotein subclasses | 0.102302643 | 6.62081E-76 |
| M_HDL_L | Lipoprotein subclasses | 0.091519924 | 2.09276E-62 |
| M_HDL_PL | Lipoprotein subclasses | 0.125561644 | 1.26E-109 |
| M_HDL_C | Lipoprotein subclasses | 0.125190573 | 1.3039E-109 |
| M_HDL_CE | Lipoprotein subclasses | 0.122858111 | 8.9196E-105 |
| M_HDL_FC | Lipoprotein subclasses | -0.053733192 | 4.94412E-24 |
| M_HDL_TG | Lipoprotein subclasses | 0.023291671 | 4.75433E-06 |
| S_HDL_P | Lipoprotein subclasses | 0.036244313 | 1.09069E-12 |
| S_HDL_C | Lipoprotein subclasses | 0.031402635 | 6.11985E-10 |
| S_HDL_CE | Lipoprotein subclasses | 0.046310675 | 4.10922E-19 |
| S_HDL_FC | Lipoprotein subclasses | -0.089778093 | 1.40557E-58 |
| S_HDL_TG | Relative lipoprotein lipid concentrations | -0.047183346 | 3.66517E-26 |
| PL_TL in CM & ELVLDL_perce | Relative lipoprotein lipid concentrations | 0.020121289 | 1.51506E-07 |
| FC_TL in CM & ELVDL_perce | Relative lipoprotein lipid concentrations | -0.034763617 | 2.43759E-16 |
| TG_TL in CM & ELVDL_perce | Relative lipoprotein lipid concentrations | -0.05174612 | 2.09601E-32 |
| PL_TL in VLVDL_perce | Relative lipoprotein lipid concentrations | 0.059701883 | 2.2833E-34 |
| C_TL in VLVDL_perce | Relative lipoprotein lipid concentrations | 0.056989667 | 1.95726E-31 |
| CE_TL in VLVDL_perce | Relative lipoprotein lipid concentrations | 0.024598597 | 6.97117E-10 |
| FC_TL in VLVDL_perce | Relative lipoprotein lipid concentrations | -0.048171166 | 2.01957E-38 |
| TG_TL in VLVDL_perce | Relative lipoprotein lipid concentrations | -0.086011693 | 3.56112E-68 |
| PL_TL in LVDL_perce | Relative lipoprotein lipid concentrations | 0.033814447 | 3.23325E-11 |
| C_TL in LVDL_perce | Relative lipoprotein lipid concentrations | 0.061398239 | 3.06982E-30 |
| CE_TL in LVDL_perce | Relative lipoprotein lipid concentrations | -0.027205861 | 2.55523E-08 |
| FC_TL in LVDL_perce | Relative lipoprotein lipid concentrations | 0.05068297 | 2.75595E-20 |
| PL_TL in MVLDL_perce | Relative lipoprotein lipid concentrations | 0.091082548 | 8.20118E-56 |
| C_TL in MVLDL_perce | Relative lipoprotein lipid concentrations | 0.088670162 | 2.75075E-52 |
| CE_TL in MVLDL_perce | Relative lipoprotein lipid concentrations | 0.078244794 | 4.76269E-44 |
| FE_TL in MVLDL_perce | Relative lipoprotein lipid concentrations | -0.091741983 | 1.008E-59 |
| TG_TL in MVLDL_perce | Relative lipoprotein lipid concentrations | 0.073834441 | 5.89698E-40 |
| PL_TL in MVLDL_perce | Relative lipoprotein lipid concentrations | 0.037981344 | 2.35299E-12 |
| C_TL in SVLDL_perce | Relative lipoprotein lipid concentrations | 0.074547844 | 9.10454E-40 |
| FC_TL in SVLDL_perce | Relative lipoprotein lipid concentrations | -0.050352994 | 8.58887E-21 |
| TG_TL in SVLDL_perce | Relative lipoprotein lipid concentrations | -0.057323046 | 3.6944E-27 |
| PL_TL in VSVLDL_perce | Relative lipoprotein lipid concentrations | 0.062686605 | 5.35038E-27 |
| C_TL in VSVLDL_perce | Relative lipoprotein lipid concentrations | 0.066324522 | 1.84589E-29 |
| CE_TL in VSVLDL_perce | Relative lipoprotein lipid concentrations | -0.055985427 | 4.96373E-23 |
| TG_TL in VSVLDL_perce | Relative lipoprotein lipid concentrations | 0.045176457 | 1.09434E-15 |
| C_TL in IDL_perce | Relative lipoprotein lipid concentrations | 0.024822166 | 7.2005E-06 |
| CE_TL in IDL_perce | Relative lipoprotein lipid concentrations | 0.056917031 | 2.23888E-26 |
| FC_TL in IDL_perce | Relative lipoprotein lipid concentrations | -0.052579174 | 1.58129E-20 |
| TG_TL in IDL_perce | Relative lipoprotein lipid concentrations | -0.045021642 | 2.46052E-18 |
| PL_TL in LLDL_perce | Relative lipoprotein lipid concentrations | 0.026766273 | 2.32533E-07 |
| C_TL in LLDL_perce | Relative lipoprotein lipid concentrations | 0.058450396 | 7.19809E-25 |
| FC_TL in LLDL_perce | Relative lipoprotein lipid concentrations | -0.038221411 | 7.29908E-12 |
| TG_TL in LLDL_perce | Relative lipoprotein lipid concentrations | 0.029416607 | 6.59951E-09 |
| PL_TL in MLDL_perce | Relative lipoprotein lipid concentrations | -0.074268968 | 1.06376E-45 |
| CE_TL in MLDL_perce | Relative lipoprotein lipid concentrations | 0.088569173 | 3.47809E-55 |
| FC_TL in MLDL_perce | Relative lipoprotein lipid concentrations | -0.022219021 | 4.4012E-05 |
| PL_TL in SLDL_perce | Relative lipoprotein lipid concentrations | 0.067819627 | 7.16885E-39 |
| CE_TL in SLDL_perce | Relative lipoprotein lipid concentrations | -0.067248107 | 6.69889E-39 |
| FC_TL in SLDL_perce | Relative lipoprotein lipid concentrations | 0.062920005 | 2.36906E-29 |
| TG_TL in SLDL_perce | Relative lipoprotein lipid concentrations | -0.069764902 | 2.47836E-36 |
| PL_TL in VLHDL_perce | Relative lipoprotein lipid concentrations | 0.025427919 | 2.11969E-06 |
| C_TL in VLHDL_perce | Relative lipoprotein lipid concentrations | -0.022187486 | 7.03767E-06 |
| CE_TL in VLHDL_perce | Relative lipoprotein lipid concentrations | 0.036912279 | 7.47669E-13 |
| FC_TL in VLHDL_perce | Relative lipoprotein lipid concentrations | -0.127031237 | 1.4981E-101 |
| TG_TL in VLHDL_perce | Relative lipoprotein lipid concentrations | -0.111693768 | 5.16489E-84 |
| PL_TL in LHDL_perce | Relative lipoprotein lipid concentrations | -0.070693291 | 1.31777E-30 |
| C_TL in LHDL_perce | Relative lipoprotein lipid concentrations | 0.078723777 | 3.05186E-42 |
| CE_TL in LHDL_perce | Relative lipoprotein lipid concentrations | 0.080924647 | 1.4962E-44 |
| FC_TL in LHDL_perce | Relative lipoprotein lipid concentrations | 0.048374273 | 4.53054E-18 |
| TG_TL in LHDL_perce | Relative lipoprotein lipid concentrations | -0.129680447 | 3.1629E-116 |
| PL_TL in MHDL_perce | Relative lipoprotein lipid concentrations | -0.115804459 | 1.96193E-92 |
| C_TL in MHDL_perce | Relative lipoprotein lipid concentrations | 0.119761456 | 1.43011E-97 |
| CE_TL in MHDL_perce | Relative lipoprotein lipid concentrations | 0.098786826 | 4.88267E-70 |
| FC_TL in MHDL_perce | Relative lipoprotein lipid concentrations | 0.140947244 | 8.0726E-124 |
| TG_TL in SHDL_perce | Relative lipoprotein lipid concentrations | -0.126183106 | 1.2071E-117 |
| C_TL in SHDL_perce | Relative lipoprotein lipid concentrations | 0.064345278 | 1.47364E-33 |
| CE_TL in SHDL_perce | Relative lipoprotein lipid concentrations | 0.037285762 | 1.38071E-12 |
| FC_TL in SHDL_perce | Relative lipoprotein lipid concentrations | 0.091948293 | 1.0225E-64 |
| TG_TL in SHDL_perce | Relative lipoprotein lipid concentrations | -0.126862349 | 8.8545E-115 |
| Analyses were adjusted for age, sex, assessment centre, Townsend Deprivation Index, educational attainment, physical activity, smoking status, sleep duration, energy intake, multivitamin use, fasting duration, body mass index, hypertension, hyperlipidemia, diabetes, cardiovascular disease, cancer, first 10 principal components of ancestry, and genotype measurement batch.  ^1^ Beta coefficients represented the difference in proteins per 1 SD higher in the MED diet scores.  ^2^ *P* below the Bonferroni-corrected thresholds (0.05/251=0.0001992032) was considered statistically significant to account for multiple comparisions. | | | |

| **Supplemental Table 4. Metabolites significantly associated with the MED diet score in elastic net regression.** | | |
| --- | --- | --- |
| **Metabolite** | **Type** | **Coefficient ^1^** |
| Spectrometer_corrected alanine | Amino acids | -0.002289219 |
| LDL_TG | Triglycerides | 0.07341357 |
| HDL_TG | Triglycerides | -0.009108884 |
| Total_PL | Phospholipids | 5.09E-06 |
| LDL_size | Lipoprotein Particle Size | 0.007004472 |
| TGvsPG_ratio | Other lipids | 0.047282139 |
| Cholines | Other lipids | 0.135039104 |
| Phosphatidylc | Other lipids | 0.005839253 |
| Sphingomyelins | Other lipids | -0.091072835 |
| Unsaturation | Fatty Acids | -0.171482895 |
| PUFA | Fatty Acids | 5.48E-05 |
| DHA | Fatty Acids | 0.099860396 |
| ω_3_Total_FA_ratio | Fatty Acids | 0.125790652 |
| PUFA_Total_FA_ratio | Fatty Acids | 0.146306733 |
| SFA_Total_FA_ratio | Fatty Acids | -0.041378266 |
| DHA_Total_FA_ratio | Fatty Acids | 0.032681021 |
| PUFA_MUFA_ratio | Fatty Acids | 0.072158768 |
| Glycine | Amino acids | 0.014863832 |
| Isoleucine | Amino acids | -0.024147441 |
| Leucine | Amino acids | 0.0083651 |
| Phenylalanine | Amino acids | -0.005683214 |
| Tyrosine | Amino acids | 0.039262657 |
| Lactate | Glycolysis related metabolites | -0.011489784 |
| Citrate | Glycolysis related metabolites | -0.002198825 |
| bOHbutyrate | Ketone bodies | 0.033268797 |
| Acetate | Ketone bodies | 0.017765801 |
| Acetone | Ketone bodies | 0.016973932 |
| Creatinine | Fluid balance | -0.072673997 |
| Albumin | Fluid balance | 0.023228427 |
| GlycA | Fluid balance | -0.073495193 |
| XXL_VLDL_P | Lipoprotein subclasses | 0.043778816 |
| XXL_VLDL_FC | Lipoprotein subclasses | 0.050219426 |
| XL_VLDL_CE | Lipoprotein subclasses | -0.057973044 |
| IDL_L | Lipoprotein subclasses | 1.05E-05 |
| S_HDL_CE | Lipoprotein subclasses | 0.01791253 |
| PL_TL in CM & ELVLDL_perce | Relative lipoprotein lipid concentrations | 0.00933367 |
| TG_TL in CM & ELVDL_perce | Relative lipoprotein lipid concentrations | 0.00486656 |
| CE_TL in VLVDL_perce | Relative lipoprotein lipid concentrations | -0.016993116 |
| FC_TL in VLVDL_perce | Relative lipoprotein lipid concentrations | 0.023403148 |
| TG_TL in VLVDL_perce | Relative lipoprotein lipid concentrations | 0.004715996 |
| PL_TL in LVDL_perce | Relative lipoprotein lipid concentrations | -0.018231495 |
| FC_TL in LVDL_perce | Relative lipoprotein lipid concentrations | 0.008728524 |
| PL_TL in MVLDL_perce | Relative lipoprotein lipid concentrations | 0.004697668 |
| CE_TL in MVLDL_perce | Relative lipoprotein lipid concentrations | 5.70E-04 |
| FE_TL in MVLDL_perce | Relative lipoprotein lipid concentrations | 0.018526845 |
| PL_TL in VSVLDL_perce | Relative lipoprotein lipid concentrations | -0.036217669 |
| CE_TL in VSVLDL_perce | Relative lipoprotein lipid concentrations | 0.008202276 |
| FC_TL in IDL_perce | Relative lipoprotein lipid concentrations | 9.02E-04 |
| PL_TL in LLDL_perce | Relative lipoprotein lipid concentrations | -0.003846847 |
| FC_TL in LLDL_perce | Relative lipoprotein lipid concentrations | 0.006638912 |
| TG_TL in LLDL_perce | Relative lipoprotein lipid concentrations | 0.071565068 |
| PL_TL in MLDL_perce | Relative lipoprotein lipid concentrations | -0.009602763 |
| CE_TL in MLDL_perce | Relative lipoprotein lipid concentrations | -0.006431616 |
| PL_TL in SLDL_perce | Relative lipoprotein lipid concentrations | 0.009383121 |
| FC_TL in SLDL_perce | Relative lipoprotein lipid concentrations | 0.005929511 |
| PL_TL in VLHDL_perce | Relative lipoprotein lipid concentrations | -0.010910656 |
| C_TL in VLHDL_perce | Relative lipoprotein lipid concentrations | 0.002123861 |
| TG_TL in VLHDL_perce | Relative lipoprotein lipid concentrations | -0.055185434 |
| PL_TL in LHDL_perce | Relative lipoprotein lipid concentrations | -0.008671967 |
| FC_TL in LHDL_perce | Relative lipoprotein lipid concentrations | -0.003281776 |
| TG_TL in LHDL_perce | Relative lipoprotein lipid concentrations | -0.02225631 |
| C_TL in MHDL_perce | Relative lipoprotein lipid concentrations | -0.029921419 |
| CE_TL in MHDL_perce | Relative lipoprotein lipid concentrations | -6.94E-04 |
| TG_TL in SHDL_perce | Relative lipoprotein lipid concentrations | -0.036460297 |
| CE_TL in SHDL_perce | Relative lipoprotein lipid concentrations | 0.026517039 |
| FC_TL in SHDL_perce | Relative lipoprotein lipid concentrations | -0.007197202 |
| ^1^ Coefficient: We applied elastic net regression (“glmnet” R package) to regress MED diet score with the candidate metabolites that significantly associated with the MED diet score using multivariable linear regression models. | | |

| **Supplemental Table 5. Association between repeated assessments of metabolic signature with RA risk ^1^.** | | | | | | | |
| --- | --- | --- | --- | --- | --- | --- | --- |
|  | Quintile 1 | Quintile 2 | Quintile 3 | Quintile 4 | Quintile 5 | Comparing the 90^th^ to 10^th^ percentiles | P for trend ^2^ |
| **Metabolic signature** |  |  |  |  |  |  |  |
| No. of RA | 30 | 24 | 28 | 24 | 20 | 126 |  |
| Person, years | 24,396 | 24,619 | 24,746 | 24,780 | 24,761 | 123,301 |  |
| Incidence per 100,000 PYs | 122.97 | 97.49 | 113.15 | 96.85 | 80.77 | 102.19 |  |
| Model 1 | 1.00 (ref) | 0.73 (0.43, 1.26) | 0.83 (0.49, 1.26) | 0.62 (0.35, 1.09) | **0.50 (0.28, 0.92)** | **0.51 (0.33, 0.80)** | 0.023 |
| Model 2 | 1.00 (ref) | 0.80 (0.46, 1.39) | 0.96 (0.56, 1.65) | 0.76 (0.42, 1.37) | 0.64 (0.34, 1.22) | 0.63 (0.38, 1.02) | 0.194 |
| Model 2+mutual adjustment | 1.00 (ref) | 0.81 (0.47, 1.40) | 0.97 (0.57, 1.67) | 0.77 (0.43, 1.40) | 0.66 (0.35, 1.27) | 0.64 (0.39, 1.05) | 0.242 |
| Abbreviations: MED, Mediterranean; RA, rheumatoid arthritis; PYs, person, years. Model 1 was adjusted for age and sex. Model 2 was additionally adjusted for assessment center, Townsend Deprivation Index, educational attainment, physical activity, smoking status, sleep duration, energy intake, multivitamin use, fasting duration, body mass index, hypertension, hyperlipidemia, diabetes, cardiovascular disease, cancer, polygenic risk score for rheumatoid arthritis, the top 10 genetic principal components, and genotype batch. Model 2 + mutual adjustment further included both the MED diet score and its metabolic signature to assess their independent associations. ^1^ Data are hazard ratios (95% confidence intervals). ^2^ *P* for trend was calculated across quintile using multivariable Cox regression models. A two-sided *P* < 0.05 was considered statistically significant. | | | | | | | |

| **Supplemental Table 6. Association between PRS and risk of RA ^1^.** | | | | |
| --- | --- | --- | --- | --- |
|  | Low PRS | Medium PRS | High PRS | Per 1-point increment |
| No. of RA cases | 144 | 642 | 337 | 1,123 |
| Person years | 251,618 | 753,423 | 250,625 | 1,255,665 |
| Incidence per 100,000 PYs | 57.23 | 85.21 | 134.46 | 89.43 |
| Model 1 | 1.00 (ref) | 1.49 (1.24, 1.78) | 2.35 (1.94, 2.86) | 1.37 (1.29, 1.45) |
| Model 2 | 1.00 (ref) | 1.48 (1.24, 1.78) | 2.33 (1.92, 2.84) | 1.36 (1.29, 1.44) |
| Abbreviations: PRS, polygenic risk score; RA, rheumatoid arthritis; PYs, person-years. Model 1 was adjusted for age and sex. Model 2 was additionally adjusted for assessment center, Townsend Deprivation Index, educational attainment, physical activity, smoking status, sleep duration, energy intake, multivitamin use, fasting duration, body mass index, hypertension, hyperlipidemia, diabetes, cardiovascular disease, cancer, polygenic risk score for rheumatoid arthritis, the top 10 genetic principal components, and genotype batch. ^1^ Data are hazard ratios (95% confidence intervals). | | | | |

| **Supplemental Table 7. Subgroup analysis of the association between MED diet score and the risk of RA by genetic risk ^1^.** | | | | | | | |
| --- | --- | --- | --- | --- | --- | --- | --- |
|  | Quintile 1 | Quintile 2 | Quintile 3 | Quintile 4 | Quintile 5 | Comparing the 90^th^ to 10^th^ percentiles | *P* for trend ^2^ |
| **Low PRS** |  |  |  |  |  |  |  |
| No. of RA cases | 42 | 37 | 26 | 23 | 16 | 144 |  |
| Person years | 60,980 | 51,022 | 56,010 | 43,661 | 39,943 | 251,618 |  |
| Incidence per 100,000 PYs | 68.87 | 72.52 | 46.42 | 52.68 | 40.06 | 57.23 |  |
| Model 1 | 1.00 (ref) | 1.00 (0.64, 1.56) | 0.61 (0.37, 1.00) | 0.67 (0.40, 1.12) | 0.50 (0.28, 0.90) | 0.53 (0.35, 0.81) | 0.004 |
| Model 2 | 1.00 (ref) | 1.11 (0.70, 1.73) | 0.72 (0.44, 1.19) | 0.85 (0.50, 1.43) | 0.68 (0.38, 1.24) | 0.68 (0.46, 1.02) | 0.116 |
| **Medium PRS** |  |  |  |  |  |  |  |
| No. of RA cases | 187 | 142 | 143 | 90 | 80 | 642 |  |
| Person years | 179,369 | 158,715 | 167,752 | 127,936 | 119,650 | 753,423 |  |
| Incidence per 100,000 PYs | 104.25 | 89.47 | 85.25 | 70.35 | 66.86 | 85.21 |  |
| Model 1 | 1.00 (ref) | 0.82 (0.66, 1.02) | 0.76 (0.61, 0.94) | 0.61 (0.47, 0.79) | 0.57 (0.44, 0.74) | 0.62 (0.51, 0.70) | <0.001 |
| Model 2 | 1.00 (ref) | 0.88 (0.70, 1.09) | 0.84 (0.67, 1.05) | 0.70 (0.54, 0.90) | 0.68 (0.52, 0.88) | 0.71 (0.59, 0.86) | 0.001 |
| **High PRS** |  |  |  |  |  |  |  |
| No. of RA cases | 81 | 88 | 73 | 58 | 37 | 337 |  |
| Person years | 60,299 | 53,452 | 55,294 | 43,189 | 38,391 | 250,625 |  |
| Incidence per 100,000 PYs | 134.33 | 164.63 | 132.02 | 134.29 | 96.38 | 134.46 |  |
| Model 1 | 1.00 (ref) | 1.16 (0.85, 1.56) | 0.92 (0.67, 1.26) | 0.90 (0.64, 1.26) | 0.64 (0.43, 0.94) | 0.72 (0.55, 0.92) | 0.010 |
| Model 2 | 1.00 (ref) | 1.20 (0.89, 1.63) | 0.97 (0.71, 1.34) | 0.98 (0.69, 1.38) | 0.69 (0.47, 1.04) | 0.77 (0.60, 1.01) | 0.055 |
| Abbreviations: MED, Mediterranean; RA, rheumatoid arthritis; PRS, polygenic risk score; PYs, person-years. Model 1 was adjusted for age and sex. Model 2 was additionally adjusted for assessment center, Townsend Deprivation Index, educational attainment, physical activity, smoking status, sleep duration, energy intake, multivitamin use, fasting duration, body mass index, hypertension, hyperlipidemia, diabetes, cardiovascular disease, cancer, polygenic risk score for rheumatoid arthritis, the top 10 genetic principal components, and genotype batch. ^a^ Data are hazard ratios (95% confidence intervals). ^b^ *P* for trend was calculated across quintile using multivariable Cox regression models. A two-sided *P* < 0.05 was considered statistically significant. | | | | | | | |

| **Supplemental Table 8. Subgroup analysis of the association between metabolic signature and the risk of RA by genetic risk ^1^.** | | | | | | | |
| --- | --- | --- | --- | --- | --- | --- | --- |
|  | Quintile 1 | Quintile 2 | Quintile 3 | Quintile 4 | Quintile 5 | Comparing the 90^th^ to 10^th^ percentiles | *P* for trend ^2^ |
| **Low PRS** |  |  |  |  |  |  |  |
| No. of RA cases | 41 | 28 | 26 | 25 | 24 | 144 |  |
| Person years | 49,700 | 48,330 | 51,594 | 50,835 | 51,159 | 251,618 |  |
| Incidence per 100,000 PYs | 82.50 | 57.94 | 50.39 | 49.18 | 46.91 | 57.23 |  |
| Model 1 | 1.00 (ref) | 0.59 (0.37, 0.96) | 0.44 (0.26, 0.72) | 0.40 (0.24, 0.66) | 0.34 (0.20, 0.58) | 0.35 (0.23, 0.54) | <0.001 |
| Model 2 | 1.00 (ref) | 0.74 (0.46, 1.21) | 0.63 (0.37, 1.06) | 0.66 (0.38, 1.14) | 0.65 (0.36, 1.117) | 0.61 (0.37, 1.01) | 0.214 |
| **Medium PRS** |  |  |  |  |  |  |  |
| No. of RA cases | 164 | 142 | 130 | 111 | 95 | 642 |  |
| Person years | 148,017 | 151.087 | 152,035 | 151,500 | 150,784 | 753,423 |  |
| Incidence per 100,000 PYs | 110.80 | 93.99 | 85.51 | 73.27 | 60.00 | 85.21 |  |
| Model 1 | 1.00 (ref) | 0.75 (0.59, 0.94) | 0.63 (0.49, 0.79) | 0.47 (0.37, 0.61) | 0.39 (0.30, 0.50) | 0.43 (0.36, 0.53) | <0.001 |
| Model 2 | 1.00 (ref) | 0.83 (0.66, 1.04) | 0.75 (0.59, 0.96) | 0.60 (0.46, 0.78) | 0.52 (0.39, 0.69) | 0.56 (0.45, 0.70) | <0.001 |
| **High PRS** |  |  |  |  |  |  |  |
| No. of RA cases | 81 | 74 | 70 | 60 | 52 | 337 |  |
| Person years | 50,829 | 51,419 | 48,235 | 49,769 | 50,373 | 250,625 |  |
| Incidence per 100,000 PYs | 159.36 | 143.92 | 145.12 | 120.56 | 103.23 | 134.46 |  |
| Model 1 | 1.00 (ref) | 0.84 (0.62, 1.15) | 0.75 (0.54, 1.04) | 0.55 (0.39, 0.78) | 0.46 (0.32, 0.66) | 0.53 (0.40, 0.70) | <0.001 |
| Model 2 | 1.00 (ref) | 0.87 (0.64, 1.20) | 0.84 (0.60, 1.17) | 0.64 (0.44, 0.92) | 0.57 (0.38, 0.83) | 0.66 (0.48, 0.89) | 0.018 |
| Abbreviations: RA, rheumatoid arthritis; PRS, polygenic risk score; PYs, person-years. Model 1 was adjusted for age and sex. Model 2 was additionally adjusted for assessment center, Townsend Deprivation Index, educational attainment, physical activity, smoking status, sleep duration, energy intake, multivitamin use, fasting duration, body mass index, hypertension, hyperlipidemia, diabetes, cardiovascular disease, cancer, polygenic risk score for rheumatoid arthritis, the top 10 genetic principal components, and genotype batch. ^1^ Data are hazard ratios (95% confidence intervals). ^2^ *P* for trend was calculated across quintile using multivariable Cox regression models. A two-sided *P* <0.05 was considered statistically significant. | | | | | | | |

| **Supplemental Table 9**. Interaction effects between MED diet score, metabolic signature and the risk of RA by genetic risk ^1^. | | | | |
| --- | --- | --- | --- | --- |
|  | **Additive interaction** | | | **Multiplicative Interaction** |
|  | ***RERI* (95% CI)** | ***AP* (95% CI)** | ***S* (95% CI)** | **HR (95% CI)** |
| **MED diet score** | –0.005 ( –0.347, 0.336) | –0.003 (–0.183, 0.177) | 0.994 (0.681, 1.452) | 0.996 (0.973, 1.021) |
| **Metabolic signature** | –0.084 (–0.453, 0.285) | –0.041 (–0.221, 0.139) | 0.926 (0.669, 1.282) | 1.00 (0.978, 1.025) |
| Abbreviations: MED, Mediterranean; RA, rheumatoid arthritis; HR, hazard ratio; CI, confidence interval; RERl, relative excess risk due to interaction; AP, attributable proportion due to interaction; S, the synergy index.  The analysis was performed in Model 2 (adjusted with assessment center, Townsend Deprivation Index, educational attainment, physical activity, smoking status, sleep duration, energy intake, multivitamin use, fasting duration, body mass index, hypertension, hyperlipidemia, diabetes, cardiovascular disease, cancer, polygenic risk score for rheumatoid arthritis, the top 10 genetic principal components, and genotype batch).  ^1^ Groups were defined by dichotomizing the MED diet score, metabolic signature, and PRS at their mean values. The reference group was participants with high MED diet score (or metabolic signature) and low PRS. | | | | |

| **Supplemental Table 10. Associations between the recomputed MED diet score and risk of RA in the UK Biobank ^1^.** | | |
| --- | --- | --- |
| **Excluded component** | **HR (95 % CI)** | ***P* value** |
| Vegetables | 0.70 (0.60-0.81) | <0.001 |
| Fruits | 0.70 (0.60-0.81) | <0.001 |
| Olive oil | 0.72 (0.61, 0.84) | <0.001 |
| Red and processed meat | 0.73 (0.63-0.84) | <0.001 |
| White meat | 0.71 (0.61-0.83) | <0.001 |
| Butter and margarine | 0.70 (0.60-0.82) | <0.001 |
| Sugary drinks | 0.76 (0.66-0.89) | 0.001 |
| Wine | 0.70 (0.58-0.84) | <0.001 |
| Legumes | 0.68 (0.56-0.81) | <0.001 |
| Nuts | 0.68 (0.57-0.82) | <0.001 |
| Seafood | 0.66 (0.55-0.80) | <0.001 |
| Sweets and desserts | 0.66 (0.55-0.79) | <0.001 |
| Sofrito | 0.72 (0.63-0.84) | <0.001 |
| Abbreviations: MED, Mediterranean; RA, rheumatoid arthritis; PRS, polygenic risk score. Multivariable Cox models adjusted for age, sex, assessment centre, Townsend Deprivation Index, educational attainment, physical activity, smoking status, sleep duration, energy intake, multivitamin use, fasting duration, body mass index, hypertension, hyperlipidemia, diabetes, cardiovascular disease, cancer, first 10 principal components of ancestry, and genotype measurement batch. ^1^ Data are hazard ratios (95% confidence intervals) for its increment from the 10^th^ to 90^th^ percentiles . | | |

| **Supplemental Table 11. Sensitivity analyses for associations between MED diet score and risk of RA ^1^.** | | | | | | | |
| --- | --- | --- | --- | --- | --- | --- | --- |
|  | Quintile 1 | Quintile 2 | Quintile 3 | Quintile 4 | Quintile 5 | Comparing the 90^th^ to 10^th^ percentiles | *P* for trend ^2^ |
| **Sensitivity analysis 1** |  |  |  |  |  |  |  |
| MED | 1.00 (ref) | 1.01 (0.86, 1.19) | 0.89 (0.75, 1.05) | 0.81 (0.67, 0.98) | 0.71 (0.57, 0.87) | 0.75 (0.65, 0.86) | <0.001 |
| Metabolic signature | 1.00 (ref) | 0.85 (0.71, 1.01) | 0.78 (0.65, 0.94) | 0.64 (0.53, 0.79) | 0.59 (0.47, 0.73) | 0.64 (0.54, 0.75) | <0.001 |
| **Sensitivity analysis 2** |  |  |  |  |  |  |  |
| MED | 1.00 (ref) | 1.00 (0.84, 1.17) | 0.87 (0.73, 1.03) | 0.80 (0.66, 0.96) | 0.68 (0.55, 0.84) | 0.73 (0.63, 0.84) | <0.001 |
| Metabolic signature | 1.00 (ref) | 0.83 (0.70, 0.99) | 0.76 (0.64, 0.92) | 0.62 (0.51, 0.76) | 0.55 (0.45, 0.69) | 0.60 (0.51, 0.71) | <0.001 |
| **Sensitivity analysis 3** |  |  |  |  |  |  |  |
| MED | 1.00 (ref) | 0.91 (0.74, 1.13) | 0.93 (0.75, 1.15) | 0.93 (0.74, 1.17) | 0.74 (0.58, 0.95) | 0.82 (0.69, 0.97) | 0.052 |
| Metabolic signature | 1.00 (ref) | 0.86 (0.69, 1.07) | 0.81 (0.64, 1.02) | 0.65 (0.51, 0.84) | 0.65 (0.50, 0.84) | 0.67 (0.54, 0.82) | <0.001 |
| **Sensitivity analysis 4** |  |  |  |  |  |  |  |
| MED | 1.00 (ref) | 1.03 (0.83, 1.28) | 0.88 (0.70, 1.10) | 0.72 (0.56, 0.94) | 0.71 (0.54, 0.94) | 0.71 (0.58, 0.86) | 0.001 |
| Metabolic signature | 1.00 (ref) | 0.85 (0.67, 1.07) | 0.82 (0.64, 1.04) | 0.60 (0.46, 0.78) | 0.50 (0.37, 0.66) | 0.55 (0.44, 0.69) | <0.001 |
| **Sensitivity analysis 5** |  |  |  |  |  |  |  |
| MED | 1.00 (ref) | 0.94 (0.79, 1.12) | 0.86 (0.72, 1.04) | 0.74 (0.60, 0.91) | 0.67 (0.53, 0.83) | 0.71 (0.61, 0.83) | <0.001 |
| Metabolic signature | 1.00 (ref) | 0.82 (0.68, 0.98) | 0.76 (0.62, 0.93) | 0.60 (0.48, 0.74) | 0.55 (0.44, 0.69) | 0.59 (0.50, 0.71) | <0.001 |
| **Sensitivity analysis 6** |  |  |  |  |  |  |  |
| MED | 1.00 (ref) | 0.96 (0.79, 1.17) | 0.84 (0.69, 1.03) | 0.76 (0.61, 0.96) | 0.64 (0.50, 0.82) | 0.71 (0.61, 0.84) | <0.001 |
| Metabolic signature | 1.00 (ref) | 0.83 (0.67, 1.02) | 0.75 (0.61, 0.94) | 0.60 (0.47, 0.76) | 0.54 (0.42, 0.69) | 0.60 (0.49, 0.72) | <0.001 |
| **Sensitivity analysis 7** |  |  |  |  |  |  |  |
| MED | 1.00 (ref) | 1.00 (0.85, 1.18) | 0.87 (0.73, 1.03) | 0.80 (0.66, 0.97) | 0.69 (0.56, 0.85) | 0.73 (0.64, 0.84) | <0.001 |
| Metabolic signature | 1.00 (ref) | 0.84 (0.70, 1.00) | 0.77 (0.64, 0.93) | 0.63 (0.51, 0.77) | 0.56 (0.45, 0.69) | 0.61 (0.52, 0.72) | <0.001 |
| Abbreviations: MED, Mediterranean; RA, rheumatoid arthritis. ^1^ The initial model was same as the model 2 in Table 2, data are hazard ratios (95% confidence intervals) . ^2^ *P* for trend was calculated across quintile using multivariable Cox regression models. A two-sided *P* < 0.05 was considered statistically significant. Sensitivity analysis 1: further adjusted for NSAIDS usage and other autoimmune diseases.  Sensitivity analysis 2: further adjusted for waist circumference.  Sensitivity analysis 3: restricted the analysis to participants with complete data. Sensitivity analysis 4: excluded participants with less than twice dietary assessment.  Sensitivity analysis 5: excluded participants with less than 2 years of follow-up.  Sensitivity analysis 6: excluded participants with non-typical diet. Sensitivity analysis 7: Fine and Gray subdistribution methods were used to assess the association of MED diet score with RA. | | | | | | | |


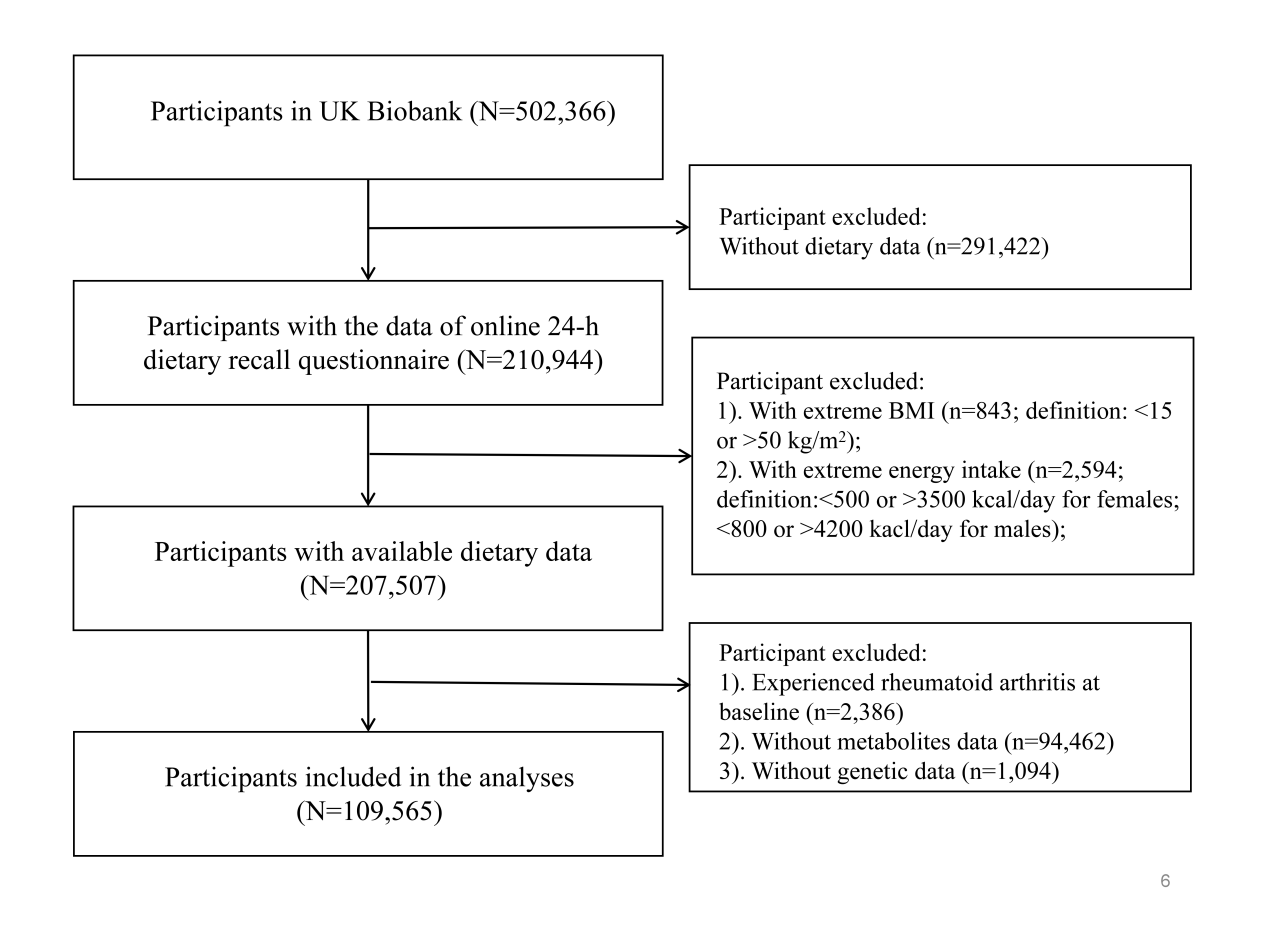


**Supplemental Figure 1. Flowchart of study participant selection.**


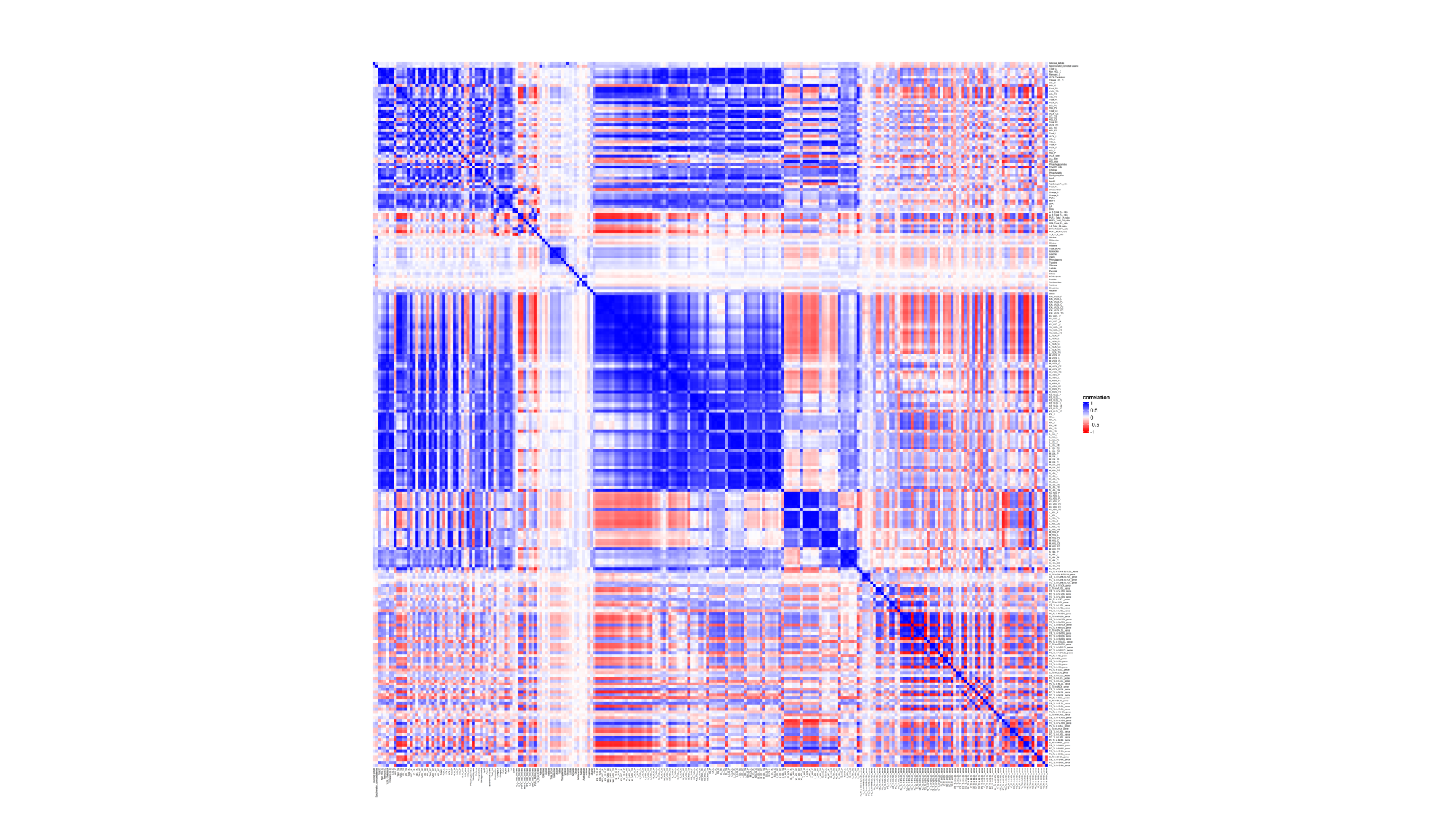


**Supplemental Figure 2. Correlation matrix for all the 251 metabolites considered in the primary analysis.**

Colors represent directions of the correlation (blue-positive and red-inverse) while the color depth indicates correlation magnitudes (the darker the stronger).


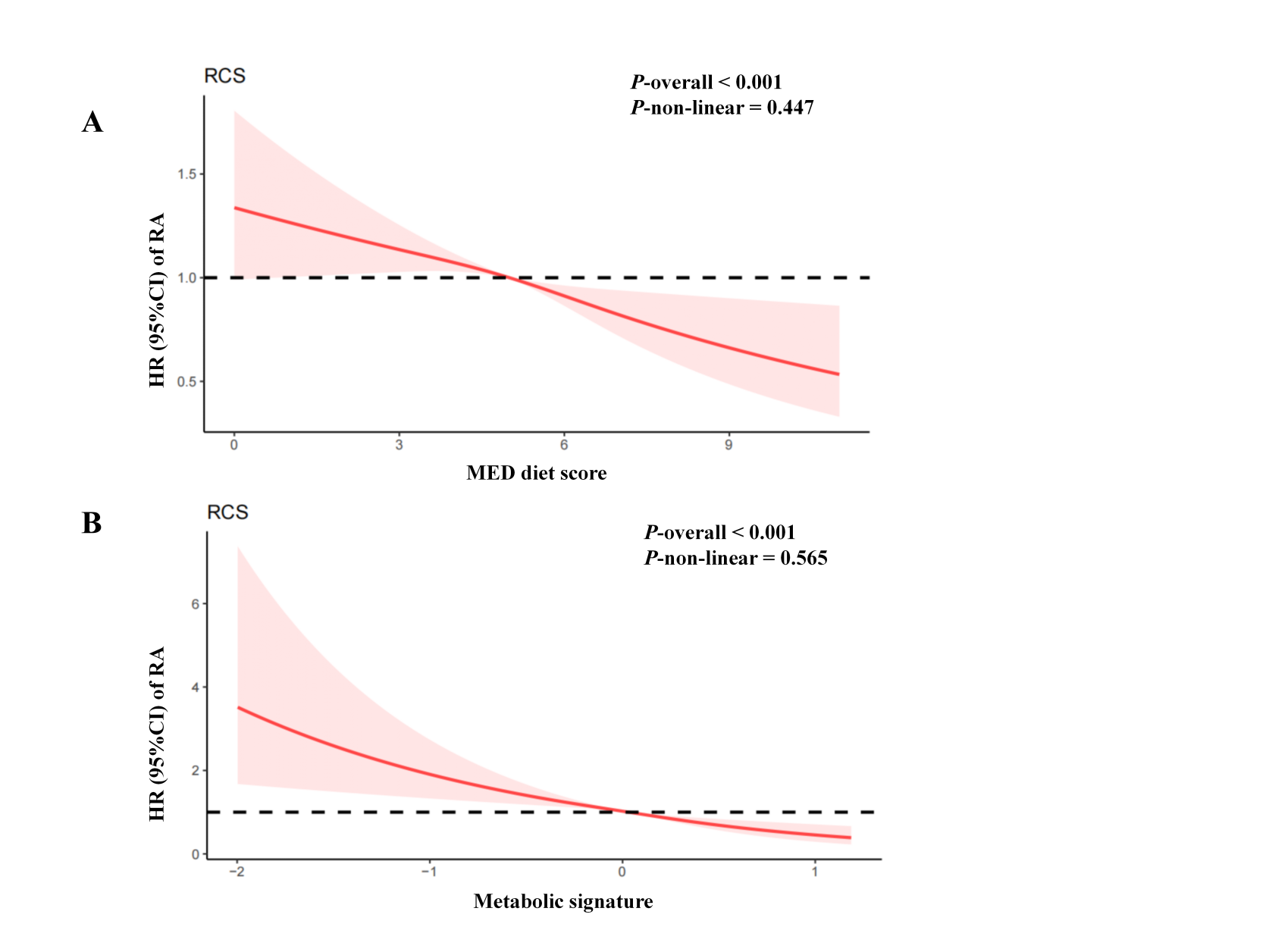


**Supplemental Figure 3. Restricted cubic spline models for the association between (A) the MED diet score, (B) metabolic signature and risk of RA.**

The 95% CIs of the adjusted HRs are represented by the shaded are. Restricted cubic spline model is adjusted the same as the Model 2 in the Table 2. A two-sided *P* < 0.05 was considered statistically significant. CI, confidence interval; HR, hazard ratio; MED, Mediterranean; RA, rheumatoid arthritis; RCS, restricted cubic spline.


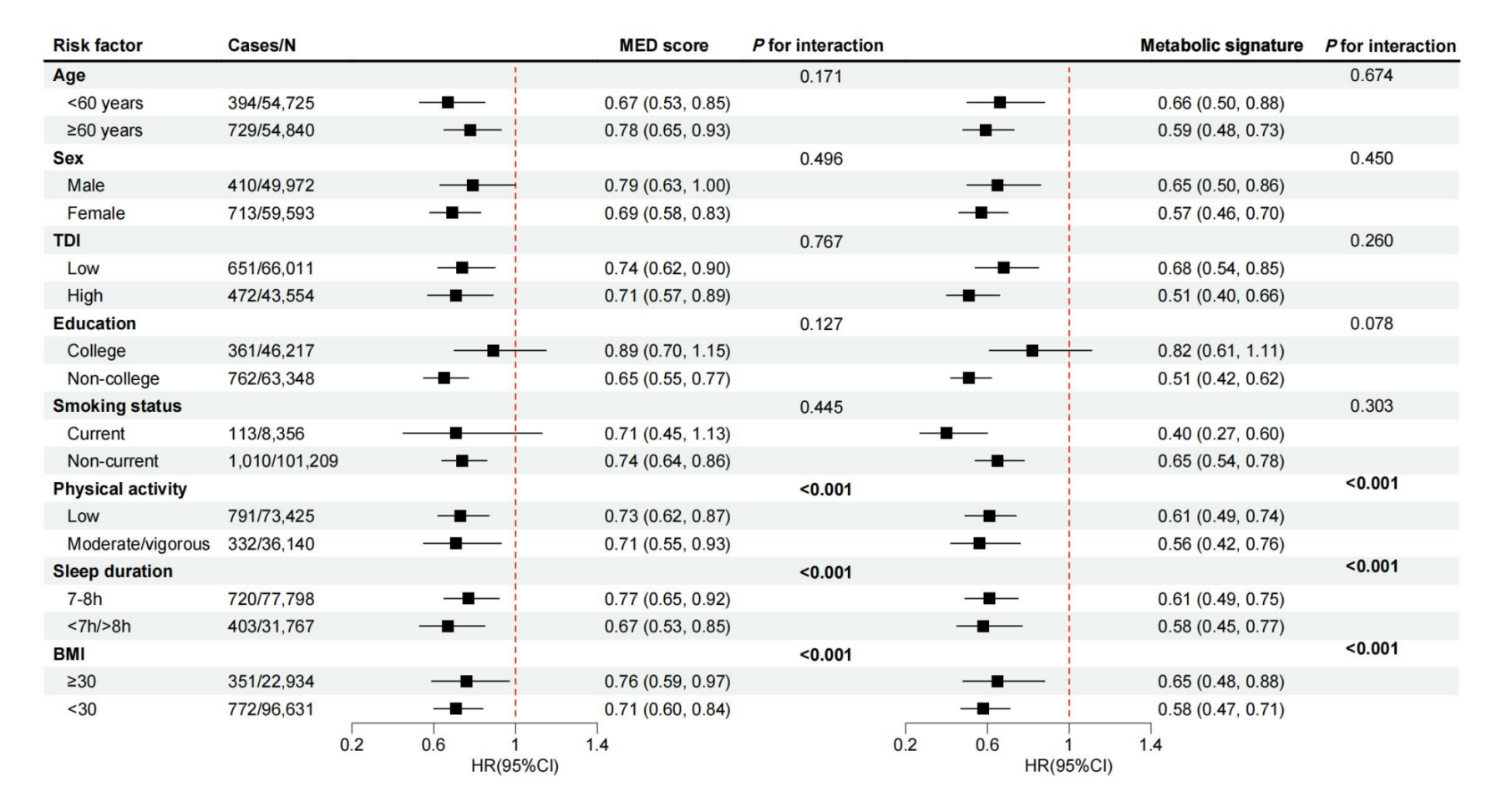


**Supplemental Figure 4. Adjusted HR (95% CI) for MED diet score, metabolic signature (comparing the 90^th^ with the 10^th^ percentiles) and risk of RA stratified by potential risk factors.**

HRs are represented by squares, and horizontal lines indicate 95% CIs. The multivariable Cox model was adjusted in the same way as Model 2 in Table 2. A two-sided *P* < 0.05 was considered statistically significant. BMI, Body mass index; CI, confidence interval; HR, hazard ratio; MED, Mediterranean; TDI, Townsend deprivation index.

**References:**

[1]Wu H, Wei J, Wang S, Chen L, Zhang J, Wang N et al. Dietary Pattern Modifies the Risk of Masld through Metabolomic Signature. Jhep Rep. 2024;6(8):101133. doi:10.1016/j.jhepr.2024.101133.

[2]Wang K, Xiang S, He Q, Liu A, Huang C, Yang Z et al. Mediterranean Diet and Associated Metabolite Signatures in Relation to Masld Progression: A Prospective Cohort Study. Hepatol Commun. 2025;9(9). doi:10.1097/HC9.00000000000007
